# Computational modelling predicts substantial carbon assimilation gains for C_3_ plants with a single-celled C_4_ biochemical pump

**DOI:** 10.1371/journal.pcbi.1007373

**Published:** 2019-09-30

**Authors:** Ivan Jurić, Julian M. Hibberd, Mike Blatt, Nigel J. Burroughs

**Affiliations:** 1 Warwick Systems Biology Centre, University of Warwick, Coventry, United Kingdom; 2 Department of Plant Sciences, University of Cambridge, Cambridge, United Kingdom; 3 Laboratory of Plant Physiology and Biophysics, University of Glasgow, Glasgow, United Kingdom; University of Uppsala, SWEDEN

## Abstract

Achieving global food security for the estimated 9 billion people by 2050 is a major scientific challenge. Crop productivity is fundamentally restricted by the rate of fixation of atmospheric carbon. The dedicated enzyme, RubisCO, has a low turnover and poor specificity for CO_2_. This limitation of C_3_ photosynthesis (the basic carbon-assimilation pathway present in all plants) is alleviated in some lineages by use of carbon-concentrating-mechanisms, such as the C_4_ cycle—a biochemical pump that concentrates CO_2_ near RubisCO increasing assimilation efficacy. Most crops use only C_3_ photosynthesis, so one promising research strategy to boost their productivity focuses on introducing a C_4_ cycle. The simplest proposal is to use the cycle to concentrate CO_2_ inside individual chloroplasts. The photosynthetic efficiency would then depend on the leakage of CO_2_ out of a chloroplast. We examine this proposal with a 3D spatial model of carbon and oxygen diffusion and C_4_ photosynthetic biochemistry inside a typical C_3_-plant mesophyll cell geometry. We find that the cost-efficiency of C_4_ photosynthesis depends on the gas permeability of the chloroplast envelope, the C_4_ pathway having higher quantum efficiency than C_3_ for permeabilities below 300 μm/s. However, at higher permeabilities the C_4_ pathway still provides a substantial boost to carbon assimilation with only a moderate decrease in efficiency. The gains would be capped by the ability of chloroplasts to harvest light, but even under realistic light regimes a 100% boost to carbon assimilation is possible. This could be achieved in conjunction with lower investment in chloroplasts if their cell surface coverage is also reduced. Incorporation of this C_4_ cycle into C_3_ crops could thus promote higher growth rates and better drought resistance in dry, high-sunlight climates.

## Author summary

Feeding the estimated world population of 9 billion people by 2050 presents a major challenge. Crop yields currently increase by about 1% each year. They would need to grow almost twice as fast to ensure global food security. New technologies that boost plant productivity are needed. A fundamental factor limiting plant growth is the speed with which plants can carry out photosynthesis. Few plants have evolved additional ‘pre-processing’ steps—the C_4_-cycle is one—that improve their photosynthetic efficiency and increase their drought resistance. Efforts are being made to introduce some form of C_4_-cycle into crops without it, but they encounter serious challenges: one should alter not only the plant’s biochemistry but also the anatomy of its leaves and cells to match that of C_4_-cycle plants. The question is, are all the alterations actually needed? We have used computational modelling to examine a scenario where the C_4_-cycle is introduced in an alternate way, with no anatomy changes made. We find that even this (previously thought unpromising) implementation strategy can substantially boost plant photosynthesis, and hence growth rate, especially in the case of plants in dry, high-sunlight climates.

## Introduction

Global food consumption is estimated to increase by over 70% by 2050 [1, 2]. To ensure global food security within the context of detrimental climate change it will be essential to achieve a substantial increase in agricultural productivity per hectare over the next couple of decades, combined with a switch to sustainable farming practices and a change in dietary habits [1]. Current yields increase per year of wheat and rice are 0.9% and 1% respectively [3]; however, sustained annual productivity increases of the order of 1.5-2% will be required (depending on the balance and success of other solutions) to ensure food safety [3]. As current methods of increasing yield saturate, development of new technologies that directly address the limiting factors of plant productivity is necessary [4]. The most fundamental factor limiting plant productivity, or the carbon assimilation rate, is the poor efficacy of the main CO_2_ fixing enzyme, Ribulose-1,5-bisphosphate-Carboxylase-Oxygenase (RubisCO). This enzyme evolved prior to the great oxygenation of the earths atmosphere [5] when CO_2_ was abundant, and it not only catalyses the fixation (carboxylation) of CO_2_ into sugars in the Calvin-Benson cycle, but also an oxygenation reaction using O_2_. This oxygenation reaction results in toxic compounds and removal of carbon from the Calvin-Benson cycle, which are resolved through an energetically costly chain of reactions known as photorespiration. The error rate (i.e. the relative frequency of oxygenation) in a typical C_3_ plant exceeds 20%. Attempts to improve RubisCO have met with limited success, as increasing reaction speed compromises enzyme specificity between CO_2_ and O_2_, and both of these factors affect assimilation efficiency. RubisCO thus lies on its Pareto front [6]. Attention has hence shifted to carbon concentrating mechanisms (CCM) that have evolved in several plant lineages, algae and cyanobacteria. CCMs increase the concentration of CO_2_ in RubisCO’s vicinity, thereby increasing the rate of carbon assimilation. In C_4_ plants, for instance, a highly efficient enzyme, Phospho-enol-pyruvate Carboxylase (PEPC), is used to initially fix CO_2_ (in its hydrated form, HCO_3_^-^), sequestering the carbon in an intermediary (a C_4_ acid such as malate), and releasing the CO_2_ in the proximity of RubisCO. This process, called the C_4_ cycle, is essentially a biochemical CO_2_ pump. C_4_ plants typically have more energy efficient carbon assimilation than C_3_ plants (i.e. require fewer photons to assimilate the same amount of carbon into sugars) thus making the C_4_ cycle a prime candidate for crop improvement [7]. The C_4_ cycle however consumes energy. Improving plant productivity by introducing the cycle into C_3_ crops is therefore a question of balancing the pumps’ costs against the efficacy of the pump (the leakage current) and the impact of the pump on the efficacy of RubisCO. This is a complex question, involving transport and biochemical issues within the context of a plant’s anatomy. Mathematical modelling is needed to address these issues and identify the factors determining the assimilation rate and photosynthetic efficacy.

C_4_ photosynthesis has evolved over sixty times in higher plants [8]. It typically appears in conjunction with so-called Kranz anatomy in which concentric layers of bundle sheath and then mesophyll cells cooperate in the photosynthetic process. Photosynthesis in these C_4_ plants is associated with multiple cell walls acting as diffusion barriers to CO_2_, preventing its escape and thereby boosting its concentration around RubisCO [8]. However, in a small number of species, the C_4_ cycle is contained within individual mesophyll cells (e.g. *Suaeda aralocaspica*, *Bienertia cycloptera* [9, 10]). It is thought that the spatial separation between the primary and the secondary carboxylases (PEPC of the C_4_ cycle and RubisCO) in the enlarged mesophyll cells of these plants mirrors the physical diffusion barriers found in Kranz-anatomy C_4_ plants [11]. A single-cell version of the C_4_ cycle may appear easier to engineer in C_3_ plants than the Kranz anatomy C_4_ cycle because the substantial anatomical remodelling of leaves and cellular architecture associated with Kranz anatomy could be avoided. However, even single-cell C_4_ plants feature notable modifications to the architecture of mesophyll cells, which facilitate the large spatial separation of the carboxylases [11]. Re-engineering the single-cell C_4_ intracellular architecture may thus also pose considerable challenges.

This raises the question of whether there is a workable solution that does not require substantial anatomical changes. Spatial separation between PEPC and RubisCO in single-cell C_4_ plants aids the C_4_ pump by providing increased diffusive resistance and essentially underpins C_4_ photosynthetic efficacy in these plants [12]. However, it is not clear if such cell-scale spatial separation is strictly necessary. To investigate this, we look at a hypothetical minimal C_4_ pathway operating in an unaltered C_3_ mesophyll cell geometry. The pathway would draw carbon from the cytoplasm and concentrate it within the chloroplast stroma. It would require targeted expression of the pathway enzymes in the cytoplasm and the stroma, a change in the expression of transporters in the chloroplast envelope to transport C_3_ and C_4_ acids, and a C_4_ regulatory mechanism to switch it off when energy/reductant availability is low. But no anatomical modifications. This minimal C_4_ photosynthetic system has previously been discussed by von Caemmerer and Furbank [13, 14] who modelled it within a compartmental paradigm. Their conclusions suggested that although a C_4_ cycle could result in higher CO_2_ assimilation rates, this would come at the expense of a substantially lower energetic efficiency of photosynthesis. However, this analysis assumed a relatively high conductance of the chloroplast envelope, the cell wall, and the plasmalemma (0.8 mol/bar m^2^s [13], which is at the upper end of most experimental estimates [15]). Due to a small spatial separation (∼ 1 μm) between the carboxylase and decarboxylase of the proposed C_4_ pump (which is well below the threshold separation (∼ 10 μm) for cost efficient single-cell C_4_ photosynthesis [12]) the viability of a single-cell based system would be strongly influenced by the permeability of the chloroplast envelope since this determines the CO_2_ leakage current. The results of von Caemmerer and Furbank should thus be revisited with a spatial model of photosynthesis, with a view of establishing design parameters for a C_4_ pump enhanced C_3_ plant.

We developed a spatial transport-assimilation model of steady-state photosynthesis to address this question. It focuses primarily on the effect of the intracellular geometry on the diffusive transport of photosynthetically relevant gases (O_2_, CO_2_, and its hydrated form HCO_3_^-^). The diffusion of these species is a limiting factor for both C_3_ and C_4_ photosynthesis. Light capture, ATP/NADPH production, Calvin-Benson cycle, and photorespiration are each assumed to function optimally. Linear and cyclic electron transfer are further coordinated to meet ATP/NADPH demand, but no coordination is assumed between the C_3_ and C_4_ cycles. The model is similar in some respects to the 3D model of C_3_ photosynthesis presented by Tholen and Zhu [16] (recently expanded to model Kranz-anatomy bioengineering [17]), but there are notable differences. Most importantly, we include C_4_ biochemistry, but we also explicitly treat oxygen’s kinetics and diffusion, whilst on a computational level we utilise the system’s symmetry to reduce the computational burden, permitting a thorough investigation of the parameter space. By examining how photosynthesis is affected by variation in cell geometry and biochemistry, we determine when the C_4_-pump is viable.

This paper is organised as follows. We first briefly present our **Model**, with additional mathematical details in supplement (S1 Appendix). In **Results** we examine the performance of C_3_ and C_4_ photosynthesis, profiling it in terms of the carbon assimilation rate and photon usage, across the range of possible values of relevant biophysical parameters. In some cases this addresses parameter uncertainty where there is a large spread in the values reported in the literature (e.g. the gas permeability of chloroplast envelope), in others it accounts for environmental variation (intra-leaf CO_2_ pressure) or examines possible synergy gain if a cellular feature is also modified (e.g. chloroplast size, chloroplast cell-surface coverage). We also assess the ability of a chloroplast to absorb and utilise photons for carbon assimilation—the *light-harvesting capacity*—which could limit assimilation of the proposed C_4_ system and thus attainable yields. In **Discussion** we propose a sequence of modifications to realise the predicted gains.

## Model

### The photosynthesis model

**Fig 1:**
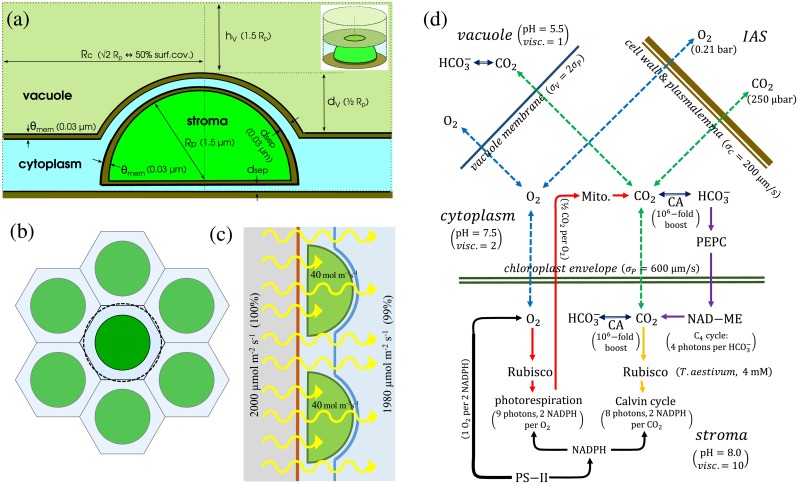
The spatial single-cell C_4_ photosynthesis model. (a): the cross-section of the simulated cylindrical volume (insert) containing a semispherically shaped chloroplast, the peripheral cytoplasm, and a part of the vacuole interior (not to scale). The cylinder radius is determined by the chloroplast surface coverage. (b): The cylindrical symmetry approximates the ‘personal’ space of an individual chloroplast in a roughly hexagonal close-packed arrangement of chloroplasts in the areas of mesophyll surface adjacent to internal airspaces. An arrangement is shown at 50% surface coverage ratio. The simulated cylinder is represented by the dashed circle. (c) A comparison of the chloroplast light-harvesting capacity expressed in terms of photon absorption per stromal volume and the fraction of maximal incoming solar flux it would correspond to. An array of a 1.5 μm radius chloroplasts with 40 mol m^-3^s^-1^ light-harvesting capacity at 50% cell surface coverage could capture 1% of maximal-insolation photon flux incident on the cell surface. (d): A schematic representation of the physical processes and chemical pathways modelled. O_2_, CO_2_, and HCO_3_^-^ can freely diffuse within individual regions, but O_2_ and CO_2_ can also diffuse through interregional boundaries (dashed green and blue arrows). Depending on the region, the interconversion of CO_2_ and HCO_3_^-^ (dark blue arrows) proceeds with or without CA assistance. CO_2_ reacting with RuBP-primed RubisCO drives the Calvin-Benson cycle (orange arrows). O_2_ reacting with RuBP-primed RubisCO activates the photorespiratory pathway (red arrows). HCO_3_^-^ reacting with PEP-primed PEPC is the starting point for the carbon transport through the C_4_ pathway (purple arrows). Oxygen production at PS-II is coupled to the NADPH consumption in the Calvin-Benson and photorespiratory cycles (black arrows). Parentheses in (a) and (d) show the default parameter values.

We use a reaction-diffusion framework to model the diffusion of CO_2_, O_2_ and HCO_3_^-^ inside a cell, solving for their position-dependent steady-state concentration profiles in order to derive photosynthetic currents. The equations are of the form

$$\begin{array}{c} D_{i}\nabla^{2}n_{i}-r_{i}(n)+s_{i}=0 \end{array}$$ (1)

where the index *i* stands for CO_2_, O_2_, and HCO_3_^-^, labelled respectively as *C*, *O*, and *B*, in the following equations. *n*_*i*_ is the spatially varying concentration of species *i*, *D*_*i*_ is the compartment-dependent diffusion coefficient, and *r*_*i*_ and *s*_*i*_ are the reaction and source terms for that species. The system is solved on a region divided into 3 compartments: chloroplast stroma, cytoplasm, and vacuole, as in Fig 1(a), with interdividing membranes modelled as low diffusion layers. Below we discuss the geometry, and the various biochemical reactions behind the reaction and source terms. Additional mathematical details are provided in S1 Appendix.

### Geometry

A typical C_3_ mesophyll cell has one large central vacuole that occupies the majority of the cell volume with other organelles and cell’s cytoplasm located around the cell’s periphery. Chloroplasts in particular, press against the cell membrane in regions adjacent to the intercellular airspace (IAS). Their density is high, with around 50%-70% of the cell surface covered by chloroplasts in a roughly hexagonal lattice arrangement (Fig 1(b)) [18, 19]. The much smaller mitochondria can move freely within the peripheral cytoplasm.

As both the sources and the sinks for CO_2_ and O_2_ are located at the cell’s periphery, the central vacuole space should play only a minor role in their transport. We therefore focus on a single, typical peripheral chloroplast and its immediate environment (the spatial region closer to this chloroplast than to its neighbours), approximating this roughly hexagonal region as a cylinder (Fig 1(a) and 1(b)) that contains one axially-centred semi-spherical chloroplast. The radius of the cylinder determines the chloroplast surface coverage fraction (the fraction of the cell surface covered with chloroplasts)—this parameter quantifies chloroplast density and thus determines the cell- and leaf- level assimilation rates. The mitochondria are mobile, so their contribution is averaged spatially and temporally at the steady state. The peripheral cytoplasm is therefore treated as a homogeneous photorespiring medium. There is little quantitative data on the precise positioning of mitochondria within the cytoplasm. Ideally, mitochondria would be positioned behind the chloroplasts (between a chloroplast and the vacuole), which might promote capture of photorespirated carbon. Such positioning is visible in micrographs of rice leaves [20], but the anatomy of rice mesophyll cells with their protruding chloroplasts is not typical for C_3_ plants. By assuming more evenly spread out mitochondria, we model photosynthesis under less ideal conditions.

### Transport and biochemistry

We focus on transport of three inorganic species—O_2_, CO_2_, and HCO_3_^-^. Whereas other metabolites are constrained to the liquid phase and typically do not pass through inter-compartmental boundaries except via dedicated channels, O_2_ and CO_2_ are gases and readily diffuse within and between cellular compartments, and between the cell interior and outside airspace. Because of this gaseous exchange, the efficacy of both C_3_ and C_4_ photosynthesis will essentially be determined by their diffusion dynamics. Diffusion within particular cellular compartments is affected by the local viscosity, while diffusion across the inter-compartmental barriers is characterised by barrier permeabilities. Diffusing gases enter and exit the simulated region through the cylinder end representing the inner surface of the cell membrane (Fig 1(a)). The permeability of a barrier to the diffusion of a metabolite is defined as a multiplicative factor, *σ*, connecting the current of the metabolite through the barrier (per-unit-area), *j*_*n*_, with the difference in the metabolite concentrations on the two sides of the barrier, *n*_1_ and *n*_2_, (Fick’s law),

$$\begin{array}{c} j_{n(1\to 2)}=\sigma (n_{1}-n_{2}) \end{array}$$ (2)

Note that the permeability (units of μm/s) is related to the leaf-level gas conductivity associated with the same barrier, *g*, (units of mol/bar m^2^s) as *g* = *ϕHσ*, where *H* is the Henry constant of the gas, and *ϕ* is the ratio of the barrier (i.e. mesophyll or chloroplast) surface and leaf surface.

Although intracellular membranes are essentially impermeable to HCO_3_^-^, its spatial dynamic also has to be treated explicitly as it strongly couples to the CO_2_ pool in the chloroplast stroma and in the cytoplasm, where we assume carbonic anhydrase (CA) is present. The CA-assisted interconversion between CO_2_ and HCO_3_^-^ is modelled as a boost to the base pH-dependent interconversion rates, Fig 1(d).

$$\begin{array}{c} v_{C\to B,(\text{CA})}\left(\text{pH}\right)=\eta_{CA}v_{C\to B\;(\text{base})}\left(\text{pH}\right)=\eta_{CA}(k_{CO_{2}}+k_{OH-K_{w}}/10^{-\text{pH}}M) \end{array}$$ (3)

$$\begin{array}{c} v_{B\to C,(\text{CA})}\left(\text{pH}\right)=\eta_{CA}v_{B\to C\;(\text{base})}\left(\text{pH}\right)=\eta_{CA}(k_{d}\cdot 10^{-\text{pH}}M+k_{HCO_{3}^{-}}) \end{array}$$ (4)

where the *k*-factors determine the base rates of CO_2_+H_2_O↔HCO_3_^-^+H^+^ and CO_2_+OH^-^↔HCO_3_^-^ reactions [21]. The dimensionless activity factor, *η*_*CA*_, accounts for both the efficiency of CA and its concentration. The simple scaling relation is possible because the enzyme-mediated reaction is reversible, thus satisfying detailed balance (see S1 Appendix). We do not consider possible changes in compartmental pH due to HCO_3_^-^ level shifts, since the pH in cytosol and chloroplast stroma is strongly buffered by phosphates and phosphate esters, with buffer capacities in 20 − 80 mM H^+^ per pH unit range [22–24] whilst our results show that the shifts in HCO_3_^-^ concentration seldom exceed 0.2 mM (Fig A in S1 Figures).

The biochemistry of carbon assimilation is well established and has been a subject of numerous mathematical models [25–27]. It is briefly summarised and discussed in the context of our model equations in the following paragraphs.

The reaction of HCO_3_^-^ with the PEPC-bound PEP in the cytoplasm is the entry point of carbon in the C_4_ cycle. The PEP carboxylation rate determines at steady state the rate of CO_2_ release from C_4_-acid decarboxylation in the stroma. We assume that the levels of C_3_/C_4_ intermediaries are large enough not to impede carbon transfer, so that intermediary steps in the C_4_ pathway need not be explicitly modelled. The concentrations of the C_4_ enzymes involved in the these parts of the pathway are likewise assumed non-limiting and sufficient at all concentrations of cytoplasmic PEPC, which we use as a measure of C_4_ pathway expression.

The Calvin-Benson cycle’s main function is to generate glucose, a 6-carbon compound from 6 CO_2_. To sequentially increase the carbon content it uses ribulose bisphosphate (RuBP), a 5-carbon compound. RubisCO catalyses the reaction between RuBP and CO_2_ to generate two 3-phosphoglycerate molecules (3-carbon compounds) that are subsequently utilised to regenerate RuBP and generate glucose. RubisCO also catalyses a reaction between RuBP and O_2_, creating one 3-phosphoglycerate and one 2-phosphoglycolate molecule. 2-phosphoglycolate is recycled via the photorespiration pathway; for every two molecules one 3-phosphoglycerate molecule is reformed and one CO_2_ molecule is released in mitochondria. The competing RuBP carboxylation and oxygenation reactions occur in the chloroplast stroma. The oxygenation rate determines at steady state the rate of mitochondrial release of photorespirated CO_2_ in the peripheral cytoplasm. Both carboxylation and oxygenation determine the net rate of carbon assimilation. The consumption of the reductant (NADPH) by the Calvin-Benson cycle and photorespiration must be matched by its production via linear electron transfer chain. (We ignore the contribution from the mitochondrial electron transfer chain, which in lit conditions will be small in comparison.) This couples O_2_ production in the chloroplast thylakoid with RuBP carboxylation and oxygenation rates (at steady state). We initially assume RuBP is not limited, later imposing a limitation on its regeneration to reflect a light harvesting cap (ATP and NADPH then being limiting).

The reaction terms *r*_*i*_ for the three species, Eq (1), comprising all the above described processes are

$$\begin{array}{c} r_{C}\left(n\left(r\right),r\right)=\chi_{p}\left(r\right)v_{C}c_{R}\frac{n_{C}\left(r\right)}{n_{C}\left(r\right)+n_{O}\left(r\right)K_{C}/K_{O}+K_{C}}+v_{C\to B}\left(r\right)n_{C}\left(r\right)-v_{B\to C}\left(r\right)n_{B}\left(r\right) \end{array}$$ (5)

$$\begin{array}{c} r_{O}\left(n\left(r\right),r\right)=\chi_{p}\left(r\right)v_{O}c_{R}\frac{n_{O}\left(r\right)}{n_{O}\left(r\right)+n_{C}\left(r\right)K_{O}/K_{C}+K_{O}} \end{array}$$ (6)

$$\begin{array}{c} r_{B}\left(n\left(r\right),r\right)=\chi_{c}\left(r\right)v_{B}c_{P}\frac{n_{B}\left(r\right)}{n_{B}\left(r\right)+K_{B}}-v_{C\to B}\left(r\right)n_{C}\left(r\right)+v_{B\to C}\left(r\right)n_{B}\left(r\right) \end{array}$$ (7)

In the preceding equations we have used characteristic functions *χ*_*p*_(***r***), *χ*_*c*_(***r***), and *χ*_*v*_(***r***) to demarcate the spatial regions corresponding to the chloroplast (plastid) interior, the cytosol, and the vacuole interior respectively. *c*_*R*_ and *c*_*P*_ are concentrations of RuBP-primed stromal RubisCO and PEP-primed cytosolic PEPC. *v*_*i*_ and *K*_*i*_ are Michaelis-Menten parameters for the modelled enzymatic reactions.

The source terms *s*_*i*_ corresponding to photorespiratory CO_2_ release in the cytosol mitochondria, C_4_ cycle CO_2_ release inside chloroplast stroma, and photosynthetic O_2_ production on chloroplast thylakoids are given in terms of currents (at steady state)

$$\begin{array}{c} s_{C}\left(r\right)=\chi_{p}\left(r\right)\frac{J_{C4}}{V_{p}}+\chi_{c}\left(r\right)\frac{\frac{1}{2}J_{phresp}}{V_{c}^{\prime }} \end{array}$$ (8)

$$\begin{array}{c} s_{O}\left(r\right)=\chi_{p}\left(r\right)\frac{J_{Calvin}+J_{phresp}}{V_{p}} \end{array}$$ (9)

where *V*_*i*_ = ∫ *χ*_*i*_(***r***)*d*^3^***r*** and the reaction currents are defined as

$$\begin{array}{c} J_{Calvin}=\int v_{C}c_{R}\frac{n_{C}\left(r\right)}{n_{C}\left(r\right)+n_{O}\left(r\right)K_{C}/K_{O}+K_{C}}\chi_{p}\left(r\right)d^{3}r \end{array}$$ (10)

$$\begin{array}{c} J_{phresp}=\int v_{O}c_{r}\frac{n_{O}\left(r\right)}{n_{O}\left(r\right)+n_{C}\left(r\right)K_{O}/K_{C}+K_{O}}\chi_{p}\left(r\right)d^{3}r \end{array}$$ (11)

$$\begin{array}{c} J_{C4}=\int v_{B}c_{P}\frac{n_{B}\left(r\right)}{n_{B}\left(r\right)+K_{B}}\chi_{c}\left(r\right)d^{3}r \end{array}$$ (12)

### Energy input and measures

The rate of assimilation is expressed on a cell-surface-area basis. The photon cost of carbon fixation (the number of photons needed per assimilated carbon atom to cover the costs of the Calvin-Benson cycle, photorespiration, and the C_4_ cycle) is quantified assuming optimal usage of the linear and cyclic electron transfer chains [28, 29], as detailed in the following paragraph.

Linear electron transfer allows for reduction of NADP^+^ to NADPH, needed in the photorespiratory and Calvin-Benson cycle. Absorption of 4 photons will result in reduction of one NADP^+^ molecule, while also transporting 6 protons into the thylakoid lumen. The proton gradient is used to run ATP-synthase, which produces one ATP for every 4 protons exiting the lumen. The stoichiometry of the linear electron transfer perfectly matches that of the Calvin-Benson cycle, which requires 3 ATP and 2 NADPH to fix one CO_2_ molecule and regenerate the RuBP substrate. Photorespiration and the C_4_ cycle however require additional ATP (3.5 ATP and 2 NADPH per oxygenated RuBP molecule, and 2 ATP (and no NADPH) per C atom transferred via the C_4_ cycle). The energy for this additional ATP production is provided by cyclic electron transfer, which is more efficient than linear transfer at generating the proton gradient. It transfers 2 protons per photon, but does not reduce NADP^+^. Note however that these are estimates, particularly for the efficiencies of the cyclic transfer and the ATP synthase. For instance, it is not clear if the proton-to-ATP stoichiometry of a chloroplast ATP-synthase is 12:3 or 14:3 [30]. A recent work [31] has shown that while the structural (i.e. binding site) stoichiometry of the spinach chloroplast ATP-synthase is 14:3, the thermodynamic ratio is 12:3, i.e. four protons are transported per ATP. The 12:3 ratio is also commonly used in the modelling literature [28, 32], so by using this ratio in our model we ensure that the results are comparable with extant modelling literature. However, both stoichiometries produce similar results in our model (Fig B in S1 Figures). Optimal light use would thus amount to 8 photons per CO_2_ molecule fixed, 9 photons to deal with each RuBP oxygenation event, and 4 photons per carbon atom transferred by the C_4_ cycle. This optimal use requires that the plant adjusts the current through the linear and the cyclic electron chain according to need and assumes that NADPH is used predominantly for photosynthesis. The ability of C_3_ plants to adjust the balance of the linear and cyclic electron fluxes has been demonstrated experimentally [33], and modelling has suggested that such adjustments might be directed by a straightforward change in metabolite demand [34, 35].

Total energy consumption cannot exceed *the light-harvesting capacity* of chloroplasts, defined here as the combined capacity to absorb light *and* to use the absorbed energy to generate ATP and replenish NADPH (thus it encompasses both the capacity of chlorophyll antennae and the linear/cyclic electron pathways). We express the energy consumption and the light-harvesting capacity in terms of (photosynthetically active) photons absorbed per stroma volume in unit time (units of mol m^-3^s^-1^), as in Xiao *et al* [36]. We use this measure, instead of e.g. light consumption per chloroplast or per cell- or leaf- surface, because we want to examine how the changes in the chloroplast surface coverage or chloroplast size affect the photosynthetic efficiency. If the chloroplast anatomy is preserved, a unit of stromal volume will on average contain a certain fixed amount of thylakoid. Hence, the per-volume measure of light use and harvesting capacity is an accurate proxy for the required photosynthetic activity and capacity of the thylakoid. As an illustration, with the default chloroplast geometry parameters (Table 1) and 50% cell-surface coverage, a photon consumption rate of 40 mol m^-3^s^-1^ corresponds to the absorption by the chloroplast array in the peripheral cytoplasm (Fig 1c and 1d) of 20 μmol m^-2^s^-1^ of photons incident on the cell surface, which is 1% of the peak photosynthetically active solar flux (2 mmol/m^2^s [37]). This comparison however does not extrapolate easily to the leaf level, as various structures within the leaf will scatter and absorb the incoming light, so individual chloroplasts experience varied light environments [36].

**Table 1:** The list of parameters used in the model and in calculation of derived measures. Where not explicitly varied, the parameters are fixed at their default values.

Parameter	Symbol	Default value	Note
Chloroplast radius	*r*_*P*_	1.5 μm	From Ellis and Leech [18]
Chloroplast surface coverage	*ϕ*_*plas*/*cell*_	50%	From Ellis and Leech [18]
Envelope-plasmalemma / envelope-tonoplast membrane separation	*d*_*sep*_	0.03 μm	
Envelope and tonoplast membrane thickness	*θ*_*mem*_	0.03 μm	The membrane thickness is exaggerated to improve numeric convergence. It does not affect the results except through excluded volume.
Vacuole drop	*d*_*V*_	$\frac{1}{2}r_{P}$	The depth by which the chloroplast ‘projects’ into the vacuole space (see Fig 1(a)).
Vacuole height	*h*_*V*_	1.5 × *r*_*P*_	The height (along the central axis) of the simulated part of the vacuole space.
RubisCO active site concentration	*c*_*R*_	4 mM	Known range is 2 mM-5 mM [25]
PEPC active site concentration	*c*_*P*_	variable	
RubisCO carboxylation catalysis rate	*v*_*C*_	3.8 s^-1^	For *T. aestivum* from Cousins *et al* [38]
RubisCO oxygenation catalysis rate	*v*_*O*_	0.83 s^-1^	
RubisCO Michaelis concentration for CO_2_	*K*_*C*_	9.7 μM	
RubisCO Michaelis concentration for O_2_	*K*_*O*_	244 μM	
PEPC carboxylation catalysis rate	*v*_*B*_	150 s^-1^	For *Z. mays* from Kai *et al* [39]
PEPC Michaelis concentration for HCO_3_^-^	*K*_*B*_	100 μM	
CO_2_ pressure in the IAS	$p_{CO_{2}}$	250 μbar	
O_2_ pressure in the IAS	$p_{O_{2}}$	0.21 bar	
Henry constant for CO_2_ at 20°C	*H*_*C*_	38.5 mM/bar	From dissolved concentrations at 400 μbar and 210 μmbar taken from Carroll *et al* [40] and Murray and Riley [41].
Henry constant for O_2_ at 20°C	*H*_*O*_	1.36 mM/bar	
pH in chloroplast stroma		8.0	
pH in the cytoplasm		7.5	
pH within the vacuole		5.5	
CO_2_↔HCO_3_^-^ conversion rate boost due to CA	*η*_*CA*_	10^6^	Saturating, see text.
Base rate for CO_2_+H_2_O→HCO_3_^-^+H^+^ reaction	$k_{CO_{2}}$	0.037 s^-1^	From Johnson [21].
Base rate for CO_2_+OH^-^→HCO_3_^-^ reaction	$k_{OH-K_{w}}$	7.1 ⋅ 10^−11^ Ms^-1^	
Base rate for HCO_3_^-^+H^+^→CO_2_+H_2_O reaction	*k*_*d*_	7.6 ⋅ 10^4^ M^-1^s^-1^	
Base rate for HCO_3_^-^→CO_2_+OH^-^ reaction	$k_{HCO_{3}^{-}}$	1.8 ⋅ 10^−4^ s^-1^	
Combined permeability of the cell wall and plasmalemma to O_2_ and CO_2_	*σ*_*c*_	200 μm/s	Ranges in literature from 2 to 5 ⋅ 10^3^μm/s [15, 42].
Permeability of the chloroplast envelope to O_2_ and CO_2_	*σ*_*p*_	600 μm/s	Ranges in literature from 20 μm/s [43] to >3.6 cm/s [44].
Permeability of the tonoplast membrane to O_2_ and CO_2_	*σ*_*v*_	2*σ*_*p*_	Assumed to have similar properties to the membranes forming the envelope.
Permeability of the chloroplast envelope to HCO_3_^-^		1 nm/s	Essentially zero.
Permeability of the tonoplast membrane to HCO_3_^-^		2 nm/s	
Diffusion constant for CO_2_ in water	*D*_*C*,*aq*_	1800 μm^2^/s	From Mazarei and Sandall [45]
Diffusion constant for O_2_ in water	*D*_*O*,*aq*_	1800 μm^2^/s	From Mazarei and Sandall [45]
Diffusion constant for HCO_3_^-^ in water	*D*_*B*,*aq*_	1100 μm^2^/s	From Falkowski and Raven [46]
Cytoplasm viscosity relative to water	*η*_*C*_	2	As in Tholen and Zhu [16].
Stroma viscosity relative to water	*η*_*P*_	10	
Vacuole interior viscosity relative to water	*η*_*V*_	1	
Chloroplast light-harvesting capacity	LHC	Varied	Either unlimited, or 40, or 80 mol m^-3^s^-1^.
Base photon cost of RuBP regeneration	*φ*_*Calvin*_	8	From Zhu *et al* [29]
Base photorespiration photon cost	*φ*_*phresp*_	9	
Base cost of pyruvate-to-PEP conversion	*φ*_*C*4_	4	

Limited light availability or light-harvesting capacity (LHC) is modelled by iteratively scaling-down the concentrations of the substrate-primed enzymes involved in photosynthesis (i.e. RuBP-primed RubisCO and PEP-primed PEPC) if energy requirements exceed the supply limit, so that a self-consistent solution is found where photosynthetic energy use exactly matches the available light-harvesting capacity. The adjustment reflects the limited substrate availability caused by energy scarcity. The concentrations of RuBP-primed RubisCO and PEP-primed PEPC are scaled proportionally, so that the ratio of their carboxylation capacities stays fixed. This proportional scaling corresponds to a non-discriminate use of ATP by the Calvin-Benson and the C_4_ cycle, thus no coordination is assumed between the two cycles. A plant with optimised control mechanisms would be able to alter the activity of PEPC as required to improve on this performance. Our predictions would then be underestimates.

### The choice of parameters

The default parameters for geometry and biochemistry in Table 1 are derived from common wheat (*Triticum aestivum* [18, 38]), which we chose as a representative C_3_ crop. Not all parameters are well characterised however, and some reflect environmental conditions. We hence analyse the robustness of our results to these parameters, specifically how the variation in a particular parameter affects the efficacy of the proposed C_4_ pathway. This is implemented by independently varying that parameter and the activity of the C_4_ pump (i.e. the cytoplasmic PEPC level).

One of the most important, yet poorly characterised biophysical parameters is the permeability of biological barriers to CO_2_ and O_2_. Estimates of chloroplast envelope permeability range over three orders of magnitude, 10^1^ − 10^4^ μm/s [15, 47], with recent measurements indicating it likely falls within the 200 μm/s–800 μm/s range [48]. This large variation may in part be attributed to different experimental methods (some of which have been criticised), different chemical composition of the membranes, the effect of unstirred layers, and the influence of carbonic anhydrase and of pH related effects [49]. Since the chloroplast envelope is expected to have a major influence on the efficiency of carbon assimilation, we focus on varying its permeability, while keeping the combined permeability of the cell wall and plasmalemma at 200 μm/s (representing the mid-range of experimental estimates provided by Terashima *et al* [42] and Evans *et al* [15]). A key constraint on the value of the envelope permeability is reproduction of the quantum efficiency of C_3_ photosynthesis in its native geometry (⪆ 0.05, or equivalently a photon cost ≈ 20/C [50]). The permeability of the vacuole membrane is set to twice the envelope permeability, the latter being a double membrane. The effect of independently varying the vacuole membrane permeability will be shown to be negligible.

The concentration of RubisCO active sites in the stroma is kept at 4 mM. This represents the concentration of activated and RuBP-primed RubisCO, and is roughly in the middle of the known range of RubisCO active site concentration (2-5 mM [25]).

When evaluating the relative efficiency of the C_4_ cycle, we use C_3_ photosynthesis *with the same amount* of CA in the cytoplasm as the baseline for comparison. CA is known to be present in the chloroplast stroma in C_3_ plants [51, 52]. There is also some evidence of cytoplasmic CA [51, 53], although the level of its activity and its effect on photosynthesis remains unknown. Since cytoplasmic CA improves C_3_ photosynthesis slightly (see Results), using C_3_ photosynthetic performance with cytoplasmic CA as a baseline benchmark will produce more conservative estimates of the gains of an introduced C_4_ cycle. We set the default CA activity factor to *η*_*CA*_ = 10^6^, to avoid it becoming a bottleneck for the C_4_ pump (see Results). Depending on how effective the CA strain is, a CA efficacy of 10^6^ would correspond to a CA active site concentration of 0.2 mM (spinach CA [54]) or ∼ 1 mM (pea [55]).

## Results

We first examine photosynthesis without any limit on light availability, mapping light requirements. Later we examine the impact of a light-utilisation cap on our results.

### The impact of the gas permeability of the chloroplast envelope

**Fig 2:**
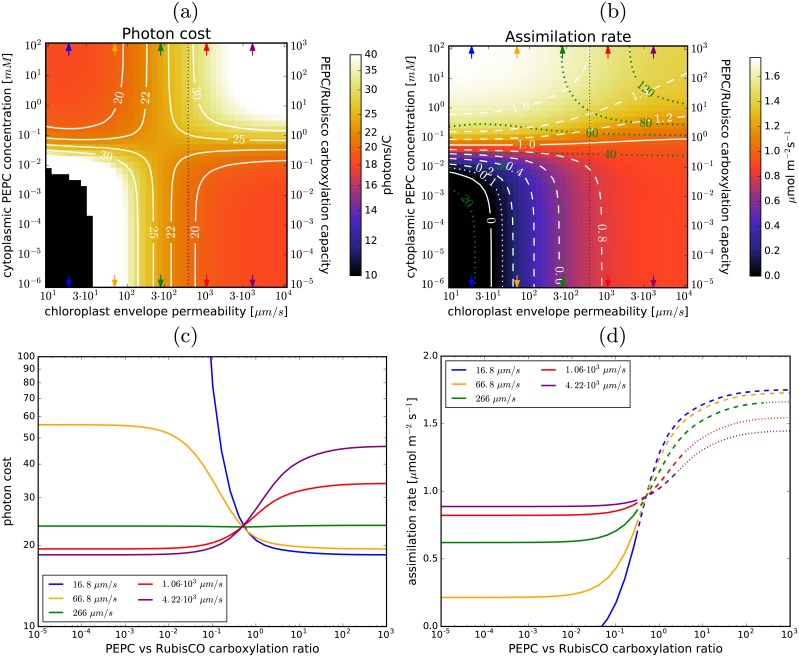
Envelope permeability and C_4_ photosynthesis. (a) and (b): The photon cost and the net assimilation rate as functions of the envelope permeability and PEPC concentration in the cytoplasm for the default parameter choice (Table 1). Level-lines are in white. The green lines in (b) mark the light-utilisation thresholds (in mol m^-3^s^-1^). In the black regions the photon cost and the assimilation rate are negative. The black vertical dotted line marks the envelope permeability used as default in other figures. Note the double y-axes for the PEPC cytoplasmic concentration and the PEPC/RubisCO carboxylation ratio. (c): Dependence of the photon cost on the PEPC-vs-Rubisco carboxylation capacity ratio for several envelope permeability values (marked with arrows in (a) and (b)). (d): The corresponding dependence of the assimilation rate. The lines become dashed (dotted) where the required light-harvesting capacity exceeds 40 mol m^-3^s^-1^ (80 mol m^-3^s^-1^).

Fig 2 shows how the photon cost and assimilation rate depend on the envelope permeability and the PEPC concentration (i.e. the pump activity). There is an envelope permeability (*σ*_*p*_) *efficacy threshold* around 300 μm/s, such that for envelope permeabilities below threshold the photon cost decreases when the pump is operational, whilst above threshold C_3_ photosynthesis is more efficient than the enhanced C_4_ system. Both photon cost and assimilation rate begin to change notably when PEPC concentration reaches 10^−2^ − 10^−1^ mM. By 1 mM PEPC, these two efficacy measures essentially saturate as the pump reaches full activity. Taking into account the volume of the chloroplast and the surrounding cytoplasm, the PEPC concentration range of 10^−2^ − 10^−1^ mM corresponds to a PEPC-to-RubisCO carboxylation capacity ratio between 0.1 and 1, while saturation occurs at ratios close to 10. By comparison, the PEPC/RubisCO activity ratio in C_4_ plants is between 2 and 6.5 [11]. Saturation in photosynthetic activity at high PEPC concentrations occurs because of a limited carbon supply—either the CA-assisted CO_2_↔HCO_3_^-^ conversion rate becomes insufficient or the diffusion of CO_2_ from internal airspaces (IAS) through the cell wall reaches its limit. The relevant rates are the CO_2_→HCO_3_^-^ conversion rate and the volume-adjusted rate of CO_2_ diffusion from IAS ($\frac{A_{C}}{V_{C}}\sigma_{c}$, where *A*_*C*_ is the cell surface area, *V*_*C*_ is the volume of the peripheral cytoplasm, and *σ*_*C*_ is the permeability of the cellular boundary). For the default choice of parameter values (including *η*_*CA*_ = 10^6^), these are roughly 4 ⋅ 10^4^ s^-1^ and 500 s^-1^, so the diffusion of CO_2_ from IAS is limiting. At *η*_*CA*_ = 10^4^ the conversion rate is only 400 s^-1^ so it becomes limiting instead. Realistically however, we can expect that energy expenditure will limit photosynthesis before that, as we demonstrate later.

Establishing whether the envelope permeability is above or below the efficacy threshold is particularly important as it determines if the C_4_ pump is more efficient than C_3_ photosynthesis. We use constraints on the efficacy of C_3_ photosynthesis (negligible PEPC concentration in Fig 2) to constrain the envelope permeability. With the cell wall and membrane permeability fixed at 20 μm/s, the photon cost of C_3_ photosynthesis reaches 20/C (known quantum efficiency of regular C_3_ photosynthesis [50]) for an envelope permeability *σ*_*p*_ ≈ 600 μm/s (Fig 2). This indicates that the permeability of the chloroplast envelope is higher than the efficacy threshold (estimated at 300 μm/s) and the photon cost of photosynthesis in a C_4_-pump enhanced cell is thus higher than for C_3_ photosynthesis alone (Fig 2(a)). We note however that the permeability efficacy threshold is dependent on CO_2_ pressure in the internal airspaces, moving to higher values as the pressure decreases (see Fig C in S1 Figures). Consequently, even for envelope permeabilities of several hundred μm/s the proposed pathway *can* become a cost-efficient strategy under conditions of CO_2_ deprivation (IAS CO_2_ pressure $p_{CO_{2}}<150$ μbar), such as may occur during prolonged stomata closure.

Although the C_4_ cycle may not be cost-effective in terms of quantum efficiency, it always increases the assimilation rate at sufficiently high PEPC activities. The assimilation gain can be substantial—up to several-fold at high PEPC concentrations—assuming photosynthesis is not limited by light (Fig 2(b)). The light harvesting capacity of chloroplasts can be estimated by examining the energy consumption of C_3_ photosynthesis when the photon cost is 20/C or less, which is the case for *σ_p_* ⪆ 600 μm/s (see Fig 2(b)). In this parameter region the light-harvesting-capacity of chloroplasts is larger than 30 mol m^-3^s^-1^ of photosynthetically active photons per stromal volume. We hence take 40 mol m^-3^s^-1^ as an estimate of the actual, or at least achievable light-harvesting capacity of an average chloroplast. Substantial assimilation gains (≥ 15%) are feasible at this light-harvesting capacity, as we demonstrate later.

**Fig 3:**
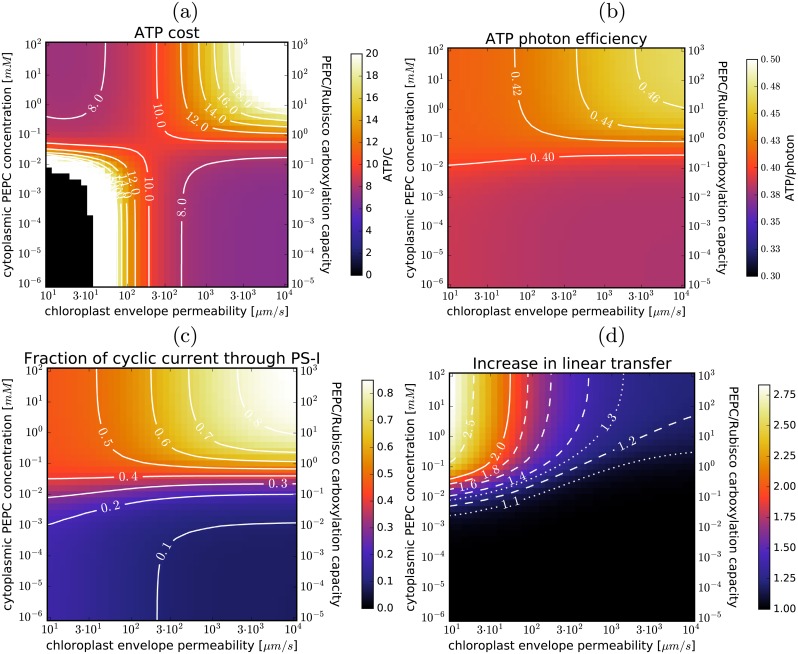
ATP use and electron transfer current. (a) ATP consumption per assimilated carbon; (b) ATP production per photon; (c) the fraction of electron current through PS-I due to cyclic transfer; and (d) the increase in the total linear electron transfer current (relative to C_3_ photosynthesis). Level-lines are in white. The dependence on the envelope permeability and PEPC concentration in the cytoplasm is shown, with the default parameter choice (same as in Fig 2).

Light use and photon cost are appropriate measures of photosynthesis costs and efficiency, since the required energy ultimately comes from sunlight. However, as explained in the **Model** section, the C_4_ cycle and the Calvin-Benson cycle do not consume ATP and NADPH in the same ratio: the C_4_ cycle does not require reductants so the ATP it requires can be provided by cyclic electron transfer. To achieve the required light-harvesting capacity we thus have to not only boost the linear electron transfer capacity (needed for Calvin-Benson cycle) but also change the balance between cyclic and linear transfer. Fig 3(a) re-expresses the results of Fig 2 in terms of ATP use. The increase in photosynthesis costs due to C_4_ cycle operation is more pronounced when expressed in ATP, but this is offset by up to 20% cheaper photon cost of ATP production when cyclic electron transfer is also used (Fig 3(b)). The cyclic transfer usage would have to increase substantially (accounting for more than 50% of PS-I current when PEPC carboxylation capacity equals that of RubisCO; Fig 3(c)). The required increase in linear electron transfer current (Fig 3(d)) is however less pronounced than the increase in light use as linear transfer is not used to supply energy to the C_4_ cycle and the latter suppresses costly RuBP oxygenation.

### The impact of other variables

*The cytoplasmic and stromal CA activity* affects the efficiency of both C_3_ and C_4_ photosynthesis. It has been conjectured that the stromal CA’s purpose is to boost CO_2_ diffusion within the chloroplast, or to facilitate CO_2_ transfer through the envelope by generating a larger CO_2_ gradient across this diffusion barrier [56]. Previous modelling has shown a minor positive impact on the assimilation rate attributable to stromal CA [16]. Our results support these findings, showing an increase to C_3_ photosynthetic efficiency and assimilation rate at CA conversion efficiencies (*η*_*CA*_) above 10^3^ (Fig D in S1 Figures). The gain reaches 10% at *η*_*CA*_ = 10^6^ and saturates at larger *η*_*CA*_. Interestingly, the effect is essentially independent of the envelope permeability value, as long as we are not close to the compensation point (where assimilation equals zero; Fig E in S1 Figures). The results are similar when CA is present both in the chloroplast stroma and in the cytoplasm, but with a somewhat larger increase in C_3_ efficiency and assimilation (∼ 14% at *η*_*CA*_ = 10^6^, Fig D in S1 Figures).

With the C_4_ cycle present, changing the efficacy of the cytoplasmic CA (keeping the efficacy of stromal CA at 10^6^) can greatly affect photosynthesis (Fig F in S1 Figures). Cytoplasmic CA activity acts as one of the bottlenecks to the pump throughput, as the C_4_ cycle uses bicarbonate (the substrate for PEPC). Hence a fast conversion of CO_2_ into HCO_3_^-^ is needed. For *η*_*CA*_ < 10^4^ the C_4_ pump is effectively non-operational and varying the PEPC level produces no noticeable change in the assimilation rate. For *η*_*CA*_ beyond 10^6^, CA ceases to be a limiting factor at PEPC concentrations below 1 mM.

The impact of *the vacuole membrane permeability* or *the thickness of the peripheral cytoplasmic layer* on the C_4_ cycle efficiency is minimal (Fig G in S1 Figures). Changing the cytoplasm thickness does change the PEPC concentration at which a particular efficiency or gain is achieved (Fig G(c)), showing that it is the ratio of PEPC-to-RubisCO activity that matters (Fig G(d)).

Changing *the permeability of the cell wall and plasmalemma* results in significant changes to the photon cost and the assimilation rate (Fig H in S1 Figures). The efficacy of the C_4_ cycle (that is, its advantage or disadvantage over C_3_ photosynthesis) is only slightly affected, however. At very high cell wall and plasmalemma permeability, the C_4_ cycle allows for a several-fold higher assimilation rate, as the bottleneck due to diffusion of CO_2_ through the cell wall is removed, but a concurrent increase in the photon cost means the chloroplast light-harvesting capacity would be limiting (this is evident from the capacity thresholds which follow the assimilation rate level-lines at high cell boundary permeability in Fig H(b) in S1 Figures).

*Diffusion of bicarbonate through the chloroplast envelope* might impact photosynthetic efficiency if the permeability of the envelope to HCO_3_^-^ is not negligible [16]. Recent experiments estimate the HCO_3_^-^ permeability between 10^-3^ and 10^-2^ μm/s [48]. We find the bicarbonate permeation has no effect on the efficacy of photosynthesis (both C_3_ and C_4_) for envelope permeabilities less than 10^-1^ μm/s, and that for permeabilities up to 10 μm/s the effect is only marginal (Fig I in S1 Figures). Bicarbonate diffusion can thus be safely neglected.

**Fig 4:**
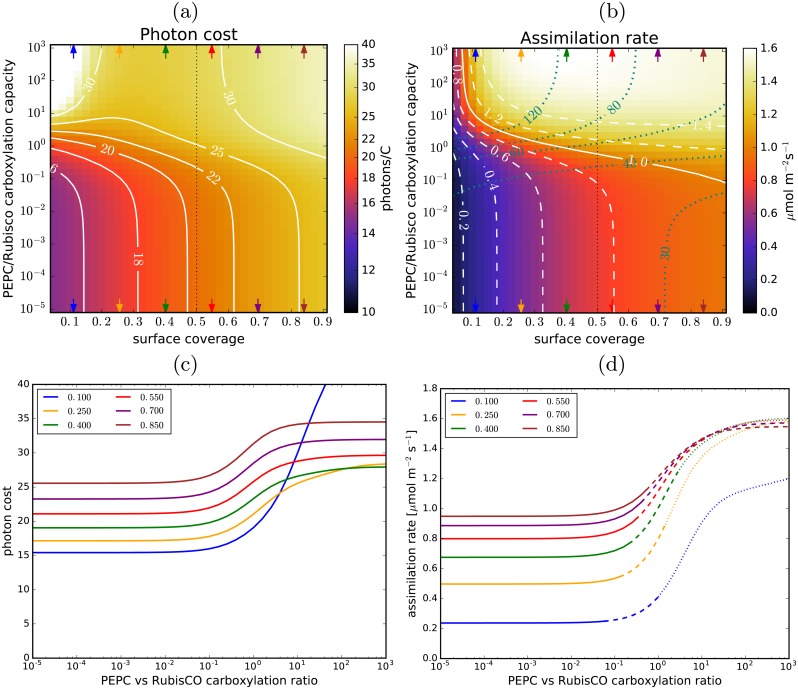
Chloroplast surface coverage and C_4_ photosynthesis. (a) and (b): The photon cost and the net assimilation rate as functions of the chloroplast surface coverage and PEPC-to-RubisCO carboxylation capacity ratio, for the default parameter choice (Table 1). Level-lines are in white. The blue lines in (b) mark the light-utilisation thresholds (in mol m^-3^s^-1^). The carboxylation capacity ratio is used instead of the PEPC concentration to quantify the C_4_ cycle activity because the cytoplasmic volume per chloroplast changes with the coverage. The black vertical dotted line marks the surface coverage used as default in other figures. (c): Dependence of the photon cost on the PEPC-vs-Rubisco carboxylation capacity ratio for several evenly-spaced surface coverage values (marked with arrows in (a) and (b)). (d): The corresponding dependence of the assimilation rate. The lines turn dashed (dotted) where the required light-harvesting capacity exceeds 40 mol m^-3^s^-1^ (80 mol m^-3^s^-1^).

Changing *the chloroplast surface coverage* (by changing the spacing between the chloroplasts while keeping their size fixed; Fig 4) alters the efficacy of the C_4_ cycle. Photon cost rises with the activation of the C_4_ pump (if the envelope permeability is above the efficacy threshold), but it also rises with surface coverage if the pump is inactive (C_3_ regime). This, coupled with the fact that the C_4_ pump provides a much stronger boost to assimilation rate at lower surface coverages (30% − 50%), leads to a remarkable and non-intuitive result that C_4_ photosynthesis allows for a higher assimilation rate *per cell surface area* (and hence *per leaf-surface area*, assuming a fixed mesophyll-to-leaf surface ratio) at lower chloroplast surface coverage, i.e. at a lower investment in chloroplasts (Fig 4(b)), while maintaining the same level of quantum efficiency.

**Fig 5:**
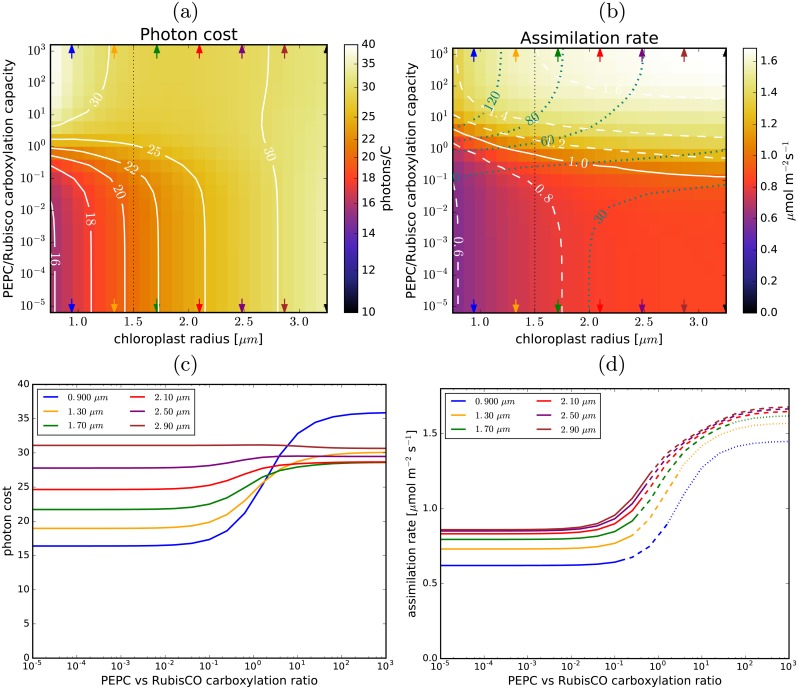
Chloroplast size and C_4_ photosynthesis. (a) and (b): The photon cost and the net assimilation rate as functions of the chloroplast radius and PEPC-to-RubisCO carboxylation capacity ratio, for the default parameter choice (Table 1). Level-lines are in white. The blue lines in (b) mark the light-utilisation thresholds (in mol m^-3^s^-1^). The carboxylation capacity ratio is used instead of the PEPC concentration to quantify the C_4_ cycle activity because the stromal volume per cell-surface area changes with chloroplast radius. The vertical dotted line marks the chloroplast size used as default in other figures. (c): Dependence of the photon cost on the PEPC-vs-Rubisco carboxylation capacity ratio for several evenly-spaced chloroplast sizes (marked with arrows in (a) and (b)). (d): The corresponding dependence of the assimilation rate. The lines turn dashed (dotted) where the required light-harvesting capacity exceeds 40 mol m^-3^s^-1^ (80 mol m^-3^s^-1^).

Increasing *the chloroplast size* (and hence RubisCO amount) while keeping the cell surface coverage constant (Fig 5) means more RubisCO per cell surface area and hence a higher assimilation rate, but also a higher photon cost because of the increased RuBP oxygenation in the case of C_3_ photosynthesis. The C_4_ cycle, at high enough PEPC concentrations, can reverse this negative trend: at PEPC-to-RubisCO capacity ratios above 3, C_4_ photosynthetic efficiency increases with chloroplast size (for very large chloroplasts C_4_ photosynthesis is even more efficient than C_3_). This results in a higher assimilation rate per cell-surface area combined with lower demands on the light-harvesting capacity (Fig 5(b)).

### The gain under limited energy availability

We now examine what gains are achievable when energy is a constraining factor. This could be either due to limited light availability or limited light-harvesting capacity. We expect that at energy inputs below the level needed to operate C_3_ photosynthesis, activating the C_4_ pump would negatively affect the assimilation rate. Therefore we consider only situations where the energy constraints do not limit C_3_ photosynthesis. This will be the case at light-utilisation caps of 40 mol m^-3^s^-1^ or more (see e.g. Fig 2(b)). If the thylakoid surface area is not the constraining factor in C_3_ photosynthesis, it should be possible to boost the chloroplast light-harvesting capacity beyond 40 mol m^-3^s^-1^ by over-expressing the photosystem complexes and associated proteins on the thylakoid (this may present a significant engineering challenge however, and there might be engineering obstacles or physical constraints forbidding a much higher light-harvesting capacity). To gain an understanding of system behaviour, we proceed with an optimistic prospect that the light-harvesting capacity can be doubled. We thus examine photosynthesis under a realistic light-utilisation cap of 40 mol m^-3^s^-1^, and under an optimistic one of 80 mol m^-3^s^-1^.

**Fig 6:**
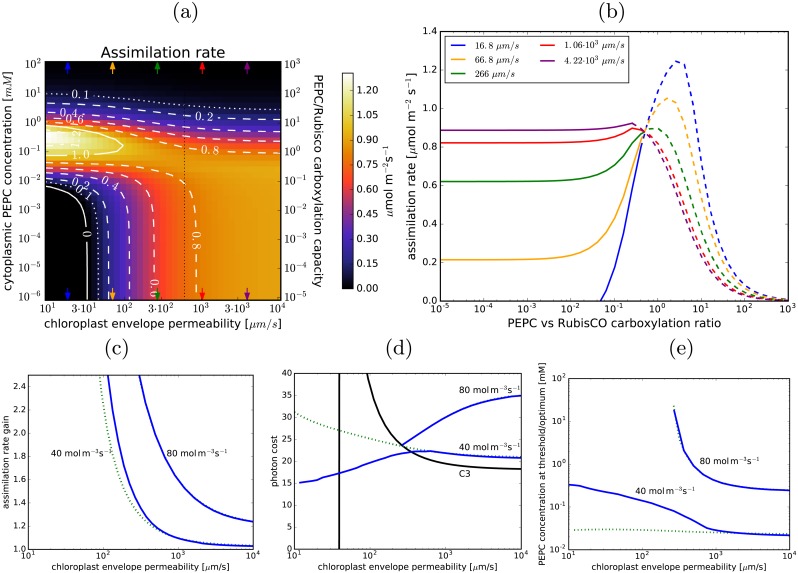
C_4_ photosynthesis at limited light-harvesting capacity. (a): The net assimilation rate as a function of the envelope permeability and PEPC concentration in the cytoplasm when the light input is capped at 40 mol m^-3^s^-1^. Parameters as in Fig 2. The vertical dotted line marks the permeability used as default in other figures. (b): The assimilation rate vs PEPC-to-Rubisco carboxylation capacity ratio for several envelope permeability values (marked with arrows in a). The lines are dashed where the light use equals the harvesting capacity. (c): The relative gain in the assimilation rate (compared to C_3_ photosynthesis) at the PEPC activity levels where the light usage reaches 40 mol m^-3^s^-1^ and 80 mol m^-3^s^-1^ (dotted green lines, corresponding to the green lines in Fig 2(b)) and the maximal assimilation gains (the maxima in panel (b)) when the corresponding light limits are imposed (blue lines). (d): the photon costs corresponding to assimilation gains in (c); the black line marks the cost of C_3_ photosynthesis (below 40 μm/s C_3_ photosynthesis cannot reach the compensation point), the blue and green lines as in (c). (e): the respective PEPC concentrations at which the optimal gains are achieved in (c) and (d).

Fig 6(a) shows how assimilation changes with the PEPC concentration at different envelope permeabilities, when the 40 mol m^-3^s^-1^ cap is imposed. The steady-state operation is not affected as long as energy use remains below the cap, so assimilation grows with C_4_ cycle activity. When energy becomes limiting, the C_4_ cycle and Calvin-Benson cycle enzymes start to compete for resources, resulting in an increase in futile cycles and reduced net assimilation at high PEPC concentrations. We might expect that the optimal assimilation under an energy constraint is then achieved exactly at the threshold where the energy usage reaches the cap. This is true for 80 mol m^-3^s^-1^ light-harvesting capacity, but not for 40 mol m^-3^s^-1^. As the C_4_ cycle changes the operating conditions in the stroma (i.e. CO_2_ levels), a situation is possible where a lower RubisCO-bound RuBP concentration (due to energy constraints) results in a higher net assimilation. The comparison of the assimilation gains (with respect to C_3_ photosynthesis) at the threshold PEPC concentration where the energy consumption reaches the cap and at the PEPC concentration where the assimilation is maximal is shown in Fig 6(c). The respective photon costs and PEPC concentrations are shown in Fig 6(d) and 6(e). It is evident that the C_4_ cycle activity has to be tuned to obtain the maximal benefit under conditions of limited and variable energy availability. Given that light supply fluctuates continually, dynamic control of the C_4_ cycle activity would have to be implemented. Alternatively, under-operating the cycle (i.e. having its activity level below the speculated optimum) may be a beneficial strategy.

Even without a fine-tuned C_4_ cycle a sizeable gain in the assimilation rate can be expected as long as envelope permeability is not too large. Looking at the photosynthetic performance at the threshold where the energy consumption reaches the 40 mol m^-3^s^-1^ cap (the green dotted line in Fig 6(c)), we predict that up to 20% gain in carbon assimilation at the envelope permeability of 600 μm/s may be achieved, with the photon cost rising by less than 10% (Fig 6(d). With 80 mol m^-3^s^-1^ capacity (and sufficient sunlight) large gains are possible over the entire range of the envelope permeability values. Assimilation could even be doubled.

**Fig 7:**
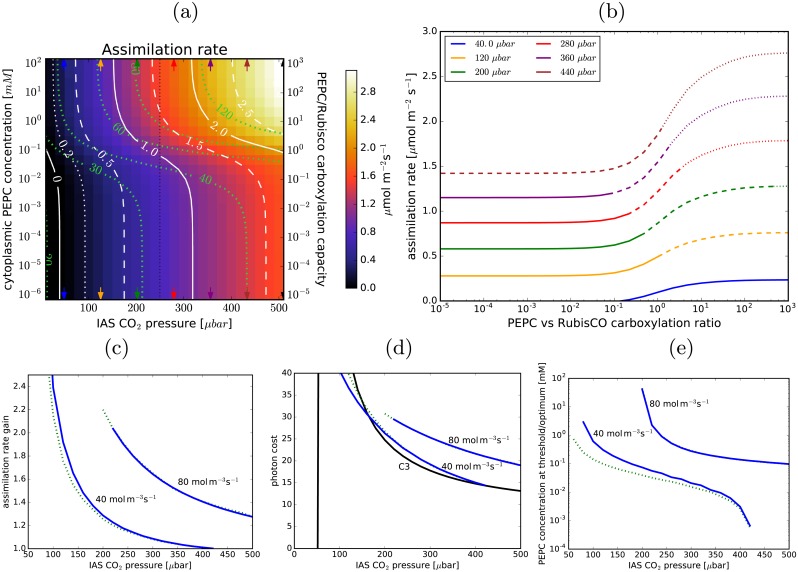
C_4_ photosynthesis at limited CO_2_ in the IAS. (a): The net assimilation rate as a function of the IAS CO_2_ pressure and PEPC concentration in the cytoplasm, for the default parameter choice (Table 1; specifically, the envelope permeability is 600 μm/s). No light utilisation cap is imposed, but the utilisation thresholds are marked in green. The vertical dotted line marks the CO_2_ pressure used as default in other figures. (b): Assimilation rate vs PEPC-to-Rubisco carboxylation capacity ratio for several CO_2_ pressures (marked with arrows in a). (c): The relative gain in the assimilation rate (compared to C_3_ photosynthesis) at the PEPC activity levels where the light usage reaches 40 mol m^-3^s^-1^ and 80 mol m^-3^s^-1^ (dotted green lines) and the maximal assimilation gains when the corresponding light limits are imposed (blue lines). (d): the photon costs corresponding to assimilation gains in (c); the black line marks the cost of C_3_ photosynthesis (below 50 μbar C_3_ photosynthesis cannot reach the compensation point), the blue and green lines as in (c). (e): the respective PEPC concentrations at which the optimal gains are achieved in (c) and (d).

Stomatal conductance is continually tuned to the environment and when conductances are low photosynthesis is frequently CO_2_ deprived. Assimilation gains from using the C_4_ pump are much more notable at low CO_2_ pressures in the intra-leaf airspaces, Fig 7(a). At 120 μbar CO_2_ the assimilation could be doubled, while still not exceeding the 40 mol m^-3^s^-1^ light-utilisation cap (Fig 7(c)). In contrast, at 400 μbar no gain is possible with that energy cap.

## Discussion

We modelled a hypothetical cytoplasm-to-stroma C_4_ cycle in a C_3_ mesophyll cell geometry, and quantified carbon assimilation and photosynthetic efficiency. The proposed C_4_ pump would lead to an increase in the assimilation rate whenever there is sufficient light-harvesting capacity and excess light is available. The magnitude of this gain is highly dependent on CO_2_ permeability of the chloroplast envelope and on operating conditions, such as the internal airspace CO_2_ pressure and light availability. At medium envelope permeability (600 μm/s), CO_2_ pressure (250 μbar), and light-harvesting capacity (40 mol m^-3^s^-1^), the gain is moderate (20%). At low CO_2_ pressure (125 μbar), or at high light availability and harvesting capacity (80 mol m^-3^s^-1^), the gain is substantial (85%), Fig 7(c). The assimilation boost comes at the price of higher photon cost (except when mesophyll is CO_2_ deprived), which may explain why this C_4_ photosynthesis strategy is not found in nature, (i.e. there is likely strong selection pressure to improve the C_4_ efficacy). Modelling the competitive evolution of single-cell vs two-cell C_4_ photosynthesis analogous to the modelling of Kranz-type C_4_ photosynthesis evolution by Heckmann *et al* [57] may provide more definite answers. Heckmann *et al* [57] determined the most likely order of mutations leading to two-cell C_4_ photosynthesis assuming a ‘greedy’ evolutionary algorithm. By also considering mutations leading to single-cell C_4_ varieties, it should be possible to establish which conditions would favour the evolution of single-cell C_4_ photosynthesis.

Due to the design of the model, which assumes optimal functioning of the C_3_/C_4_ enzymatic pathways, our predictions always represent the best case scenario. Even so, the large predicted assimilation advantage under conditions of CO_2_ deprivation is likely robust. As CO_2_ deprivation is a common hazard facing plants in dry and warm climates—which are typically well-lit—the development of the proposed C_4_ pathways could be very beneficial for creating drought-resistant high-yield crop strains. It is interesting to note that terrestrial species that *have* evolved single-celled C_4_ photosynthesis (e.g. *Suaeda aralocaspica*, *Bienertia cycloptera*) grow in salty depressions in semi-arid regions—the conditions that would likely lead to low CO_2_ within the leaf [9, 10].

Our conclusions are generally in qualitative agreement with von Caemmerer [14], but the more accurate accounting of energy use and the treatment of gas diffusion in our model produces more optimistic results. Specifically, although we agree with von Caemmerer [14] that the C_4_ cycle will be cost-inefficient, our results show the difference between carbon assimilation costs in C_3_ and C_4_ photosynthesis is smaller at lower envelope permeability or CO_2_ level, so the operation of a C_4_ cycle need not be prohibitively expensive. This means higher gains are possible as long as there remains some unused light-harvesting capacity, as Fig 6(c) demonstrates. To understand the reasons for the differences in our conclusions, we attempt a more direct comparison with the results of von Caemmerer and Furbank [13]. At 200 ppm CO_2_ in the IAS, they predict that operating the C_4_ pump at 1:1 PEPC-to-RubisCO carboxylation capacity ratio would result in a 40% increase in the assimilation rate and a 70% increase in energy cost per assimilated carbon (Fig 5 in von Caemmerer and Furbank [13]). Their model expresses gas conductances and enzyme catalytic capacities per leaf-surface area, so a comparison to our diffusion model requires an assumption of the mesophyll-to-leaf surface area ratio. For a ratio of 13.5 (similar to values observed in *A. thaliana* (8-10) [19]), the RubisCO catalytic capacities in the two models match, so we use this value for the comparison. Their conductances would then correspond to the permeabilities of the envelope, and of the cell wall and plasmalemma, of approximately 10^3^ μm/s each. With the same parameters we get a 50% increase in the assimilation rate with a 30% increase in the photon cost (from 17/C to 22/C). There is a significant difference in the predictions of the energy cost of C_4_ photosynthesis. The difference in part stems from different accounting methods. von Caemmerer and Furbank [13] considers ATP consumption whereas our quantification in terms of light-use takes into account in the fact that the C_4_ cycle does not need a reductive agent and hence its ATP requirements can be met more efficiently (up to 20%, conf. Fig 3(b)) by cyclic electron transfer. In terms of ATP we see a 50% increase in cost. The difference between this value and the 70% increase in von Caemmerer and Furbank [13] is attributable to spatial effects and diffusion.

**Fig 8:**
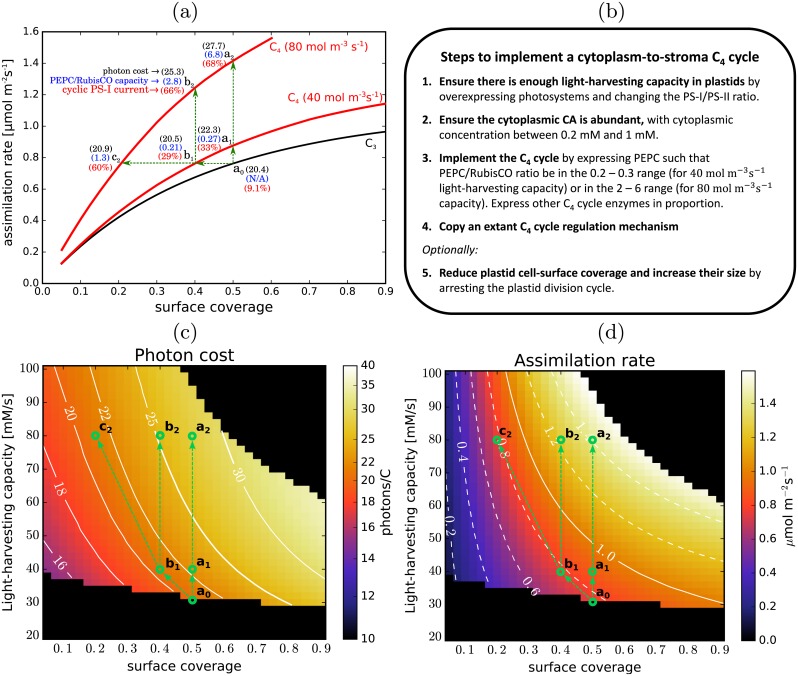
Altering chloroplast surface coverage and light harvesting capacity. (a) The assimilation rate per cell surface area as a function of chloroplast surface coverage in the case of C_3_ photosynthesis (black line) and C_4_ photosynthesis at the C_4_ cycle activity levels where light-use reaches 40 mol m^-3^s^-1^ and 80 mol m^-3^s^-1^ thresholds (red lines). The numbers in parentheses show the respective photon costs (black), PEPC-vs-RubisCO carboxylation capacity ratios (blue), and the fraction of current through PS-I due to cyclic electron transfer (red). Green arrows illustrate organism modification strategies discussed in the main text. Parameters as in Fig 4. (b) An outline of a recipe for making a functional C_4_ photosynthesising prototype. (c) and (d): The photon cost and assimilation rate as functions of the surface coverage and threshold light-use in C_4_ photosynthesis. The panels are a remapping of Fig 4 with an alternate y-axis. Level-lines are in white. In the dark region at the bottom the light-use is below the requirements of C_3_ photosynthesis. The top right dark region corresponds to PEPC levels beyond those simulated. The green circles and arrows mark the modification strategies shown in (a).

Another promising result is that the pathway’s beneficial effects can be increased further by reducing the chloroplast surface coverage, bringing it into the region in Fig 4(a) where the rise in the photon cost when the C_4_ pump is active is less pronounced. This minor change to the cell anatomy would allow for the same assimilation rate to be achieved with a reduced chloroplast investment, translating into an even higher plant growth rate. One way this could be accomplished might be to arrest or slow down the chloroplast division cycle. A possible side-effect would be an increase in the average chloroplast size, which would further benefit C_4_ photosynthesis (Fig 5). An illustration of possible benefits from a design strategy that combines the implementation of a C_4_ cycle with alterations in the chloroplast surface coverage is presented in Fig 8. The design steps are broadly outlined in Fig 8(b). Fig 8(a) shows how the assimilation rate varies with the surface coverage (assuming no changes in the chloroplast size) for C_3_ photosynthesis, and C_4_ photosynthesis at 40 mol m^-3^s^-1^ and 80 mol m^-3^s^-1^ light utilisation thresholds (compare with Fig 4(b)). Starting with C_3_ photosynthesising chloroplasts at 50% cell surface coverage (a_0_), implementing the C_4_ pump and boosting the light-harvesting capacity to 40 mol m^-3^s^-1^ (a_1_) or 80 mol m^-3^s^-1^ (a_2_) would result in a 15% or an 85% increase in the assimilation rate respectively. Alternatively, at 40 mol m^-3^s^-1^ light-harvesting capacity, the number of chloroplasts could be reduced by 20% (b_1_) without any loss in assimilation compared to C_3_ photosynthesis. Boosting the light-harvesting capacity to 80 mol m^-3^s^-1^ would allow for an even larger reduction in the number of chloroplasts while still maintaining or increasing assimilation (b_2_, c_2_). Fig 8(c) and 8(d) illustrate how the suggested modifications would move the system on the photon cost and assimilation rate landscapes. If the chloroplasts are also enlarged in the process, even larger gains may be possible. The level of required C_4_ cycle expression, quantified by the PEPC/RubisCO carboxylation capacity ratio, would not exceed the observed level of C_4_ cycle activity in C_4_ plants (2-7 [11]), even at 80 mol m^-3^s^-1^ light-harvesting capacity (Fig 8(a)). A regulation mechanism would have to be incorporated however, to moderate the activity of the C_4_ pump based on the energy availability, so as to prevent it from competing adversely with the Calvin-Benson cycle in low-light conditions. Regulation of the C_4_ cycle based on the ambient light levels and CO_2_ availability is already present in Kranz-type C_4_ plants [58], so implementing existing C_4_ regulatory mechanisms may allow this. The relative expression of the two photosystems would also need to be rebalanced, to allow for a larger cyclic electron current through PS-I (Fig 8(a)). The cyclic current would constitute ∼ 30% of total electron current through PS-I at 40 mol m^-3^s^-1^, and ∼ 60% at 80 mol m^-3^s^-1^. Such large cyclic current fractions are not commonly seen in C_3_ plants (though they are normal in C_4_ plants), however C_3_ plants can adapt to use cyclic transfer more (up to 50% of electron current through PS-I) if circumstances so require [33]. The optimal modification strategy when introducing the C_4_ cycle would be the one that maximises the return on resource investment. To calculate this however, the maintenance costs also need to be established. Quantifying the return-on-investment and deciding the optimal strategy will require additional research.

## Supporting information

S1 Figures Supplementary figures. A document containing all the supplementary figures referenced in the text.
(PDF)

S1 Appendix Supplementary model description. A more in-depth description of the model, with additional mathematical and technical details.
(PDF)

S1 Source Source code. Contains the model implementation in C++ code and a meshing algorithm in Python.
(ZIP)

S1 Dataset Raw results. The input parameter files and the raw output files used to make all the Figures.
(ZIP)
