# Supplementary material for: Computational modelling predicts substantial carbon assimilation gains for C3 plants with a single-celled C4 biochemical pump
Source: PLoS Comput Biol. 2019 Sep 30;15(9):e1007373. doi: 10.1371/journal.pcbi.1007373 (PMC6786660; doi:10.1371/journal.pcbi.1007373)

Computational modelling predicts substantial carbon assimilation gains for C_3 plants with a single-celled C_4 biochemical pump

Ivan Jurić[a,*], Julian M. Hibberd[b], Mike Blatt[c], Nigel J. Burroughs[a,*]

[a] *Warwick Systems Biology Centre, University of Warwick, Coventry, CV4 7AL, UK*
[b] *Department of Plant Sciences, University of Cambridge, Cambridge, CB2 3EA, UK*
[c] *Laboratory of Plant Physiology and Biophysics, University of Glasgow, Glasgow, G12 8QQ, UK*

Supplementary Figures

*Corresponding authors. Emails: i.juric@warwick.ac.uk, n.j.burroughs@warwick.ac.uk.

Figure A: Bicarbonate concentration and C_4 photosynthesis: (a): HCO_3^- concentration in the chloroplast stroma as function of the envelope permeability and PEPC concentration in the cytoplasm for default choice of parameter values (Table 1 in the main text). (b): the corresponding HCO_3^- concentration in the cytoplasm.

(a) (b)

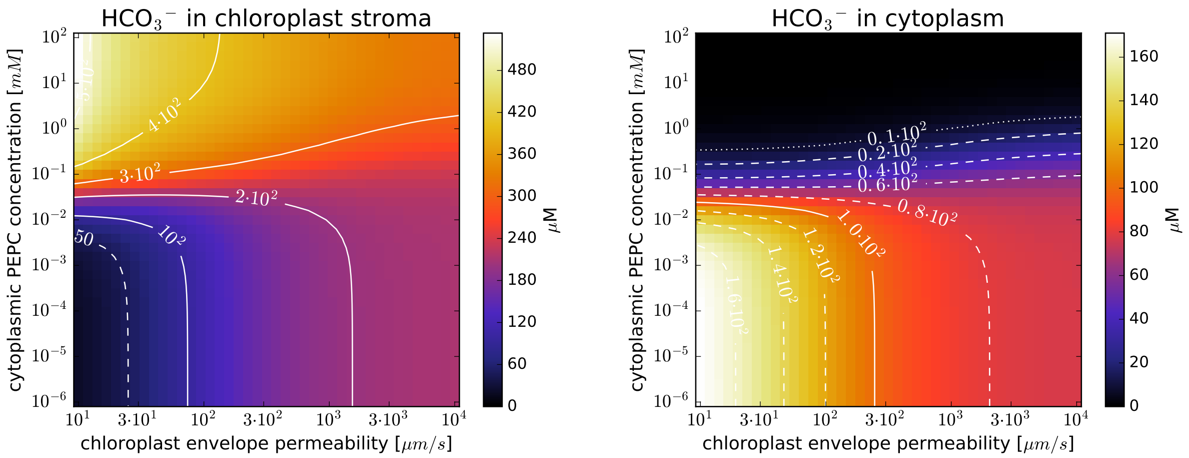

Figure B: Photon cost and ATP-synthase efficiency: (a) and (b): the photon cost as a function of the envelope permeability and PEPC concentration in the cytoplasm for two possible proton-to-ATP stoichiometries of the chloroplastic ATP synthase. (a): proton-to-ATP stoichiometry of 12:3 (the default used throughout this article); (b): proton-to-ATP stoichiometry of 14:3. The base photon costs of RuBP regeneration, photorespiration, and pyruvate-to-PEP conversion used to evaluate (b) are $\varphi_{Calvin} = 9$, $\varphi_{phresp} = 10\frac{1}{6}$, and $\varphi_{C4} = 4\frac{2}{3}$.

(a) (b)

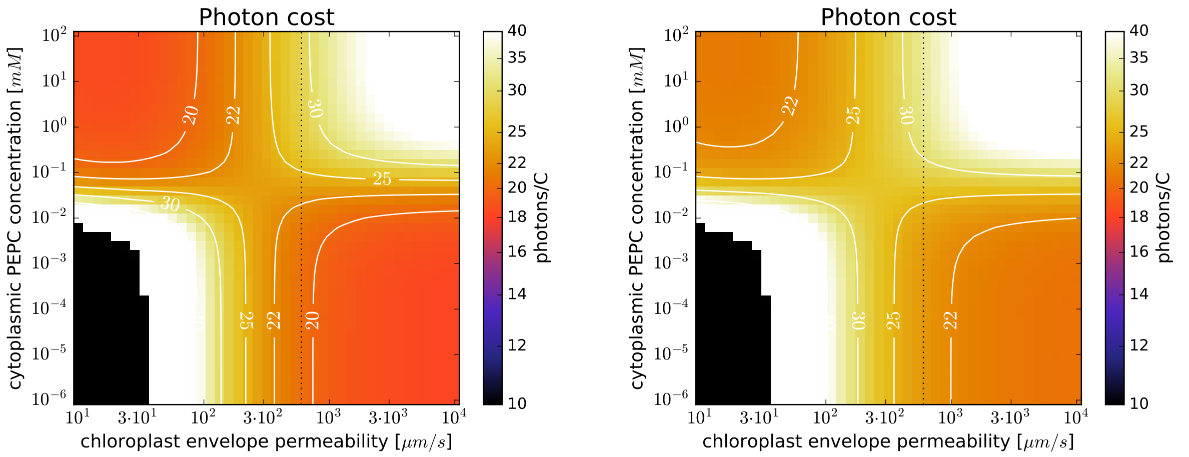

Figure C: C_4 photosynthesis at different IAS CO_2 levels: The photon cost as a function of the envelope permeability (σ_P) and PEPC concentration in the cytoplasm (c_P) at $p_{CO_2} = 150\,\mu$bar (a), $p_{CO_2} = 250\,\mu$bar (b), and $p_{CO_2} = 400\,\mu$bar (c). The dotted vertical marks the threshold envelope permeability below which the C_4 cycle is cost-efficient.

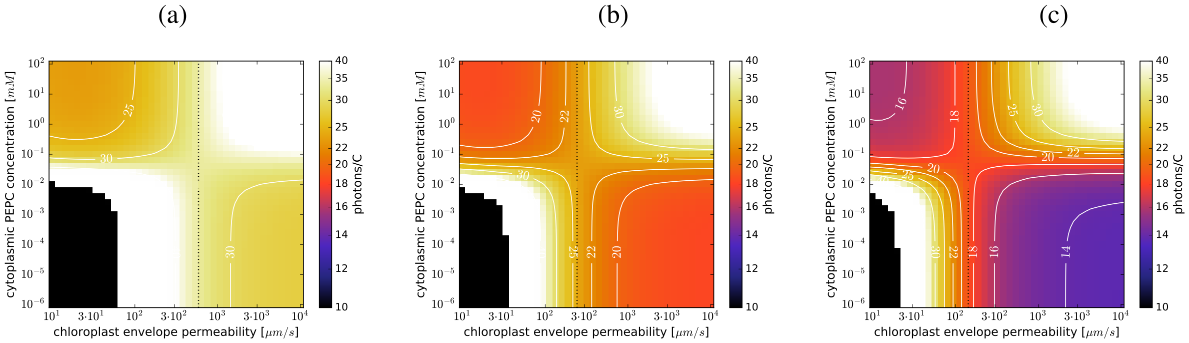

Figure D: Cytoplasmic CA and C_3 photosynthesis. The assimilation rate gain (blue) and the photon cost reduction (red) as functions of CA effectiveness (η_{CA}) for the chloroplast envelope permeability of $600\,\mu$m/s. Solid lines are for the case where CA effectiveness is increased only in the chloroplast stroma; dashed lines are for the case where CA effectiveness increases both in the stroma and in the cytoplasm. The vertical dotted line marks the boost factor used as default in other figures.

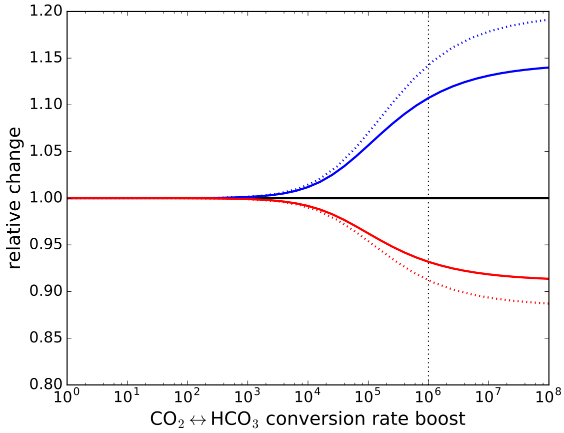

Figure E: CA efficacy vs. envelope permeability in C_3 photosynthesis. (a) and (b): Relative increase in carbon assimilation rate and reduction in photon cost due to presence of CA in the chloroplast stroma, as functions of the envelope permeability (σ_P) and the CO_2/HCO_3^- interconversion rate boost (η_{CA}); for $p_{CO_2} = 250\,\mu$bar and $\sigma_C = 200\,\mu$m/s. (c) and (d): Same as (a) and (b) for the case when CA is present both in the stroma and in the cytoplasm.

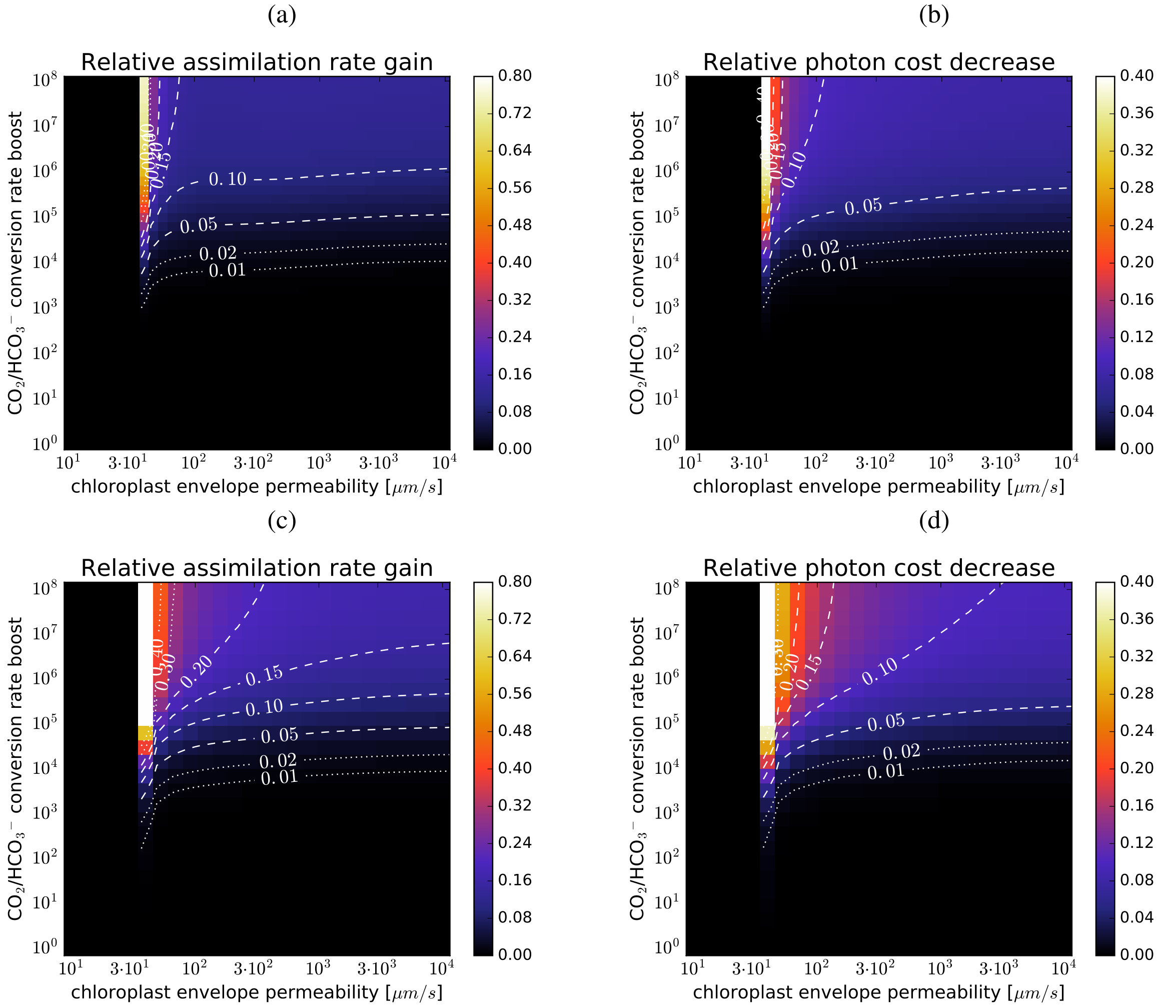

Figure F: CA efficacy and C_4 photosynthesis: (a) and (b): the photon cost and the net assimilation rate as functions of the $CO_2 \leftrightarrow HCO_3^-$ conversion rate boost due to cytoplasmic CA (η_{CA}) and PEPC concentration in the cytoplasm (c_P), for the default parameter choice (Table 1 in the main text). (c) and (d): the photon cost and the net assimilation rate as functions of the envelope permeability (σ_P) and PEPC concentration in the cytoplasm (c_P), when cytoplasmic CA is insufficient ($\eta_{CA} = 10^4$). The vertical dotted lines mark the values used as defaults in other figures. The green lines in (b) and (d) mark the light-utilisation thresholds (in mol m^{-3}s^{-1}).

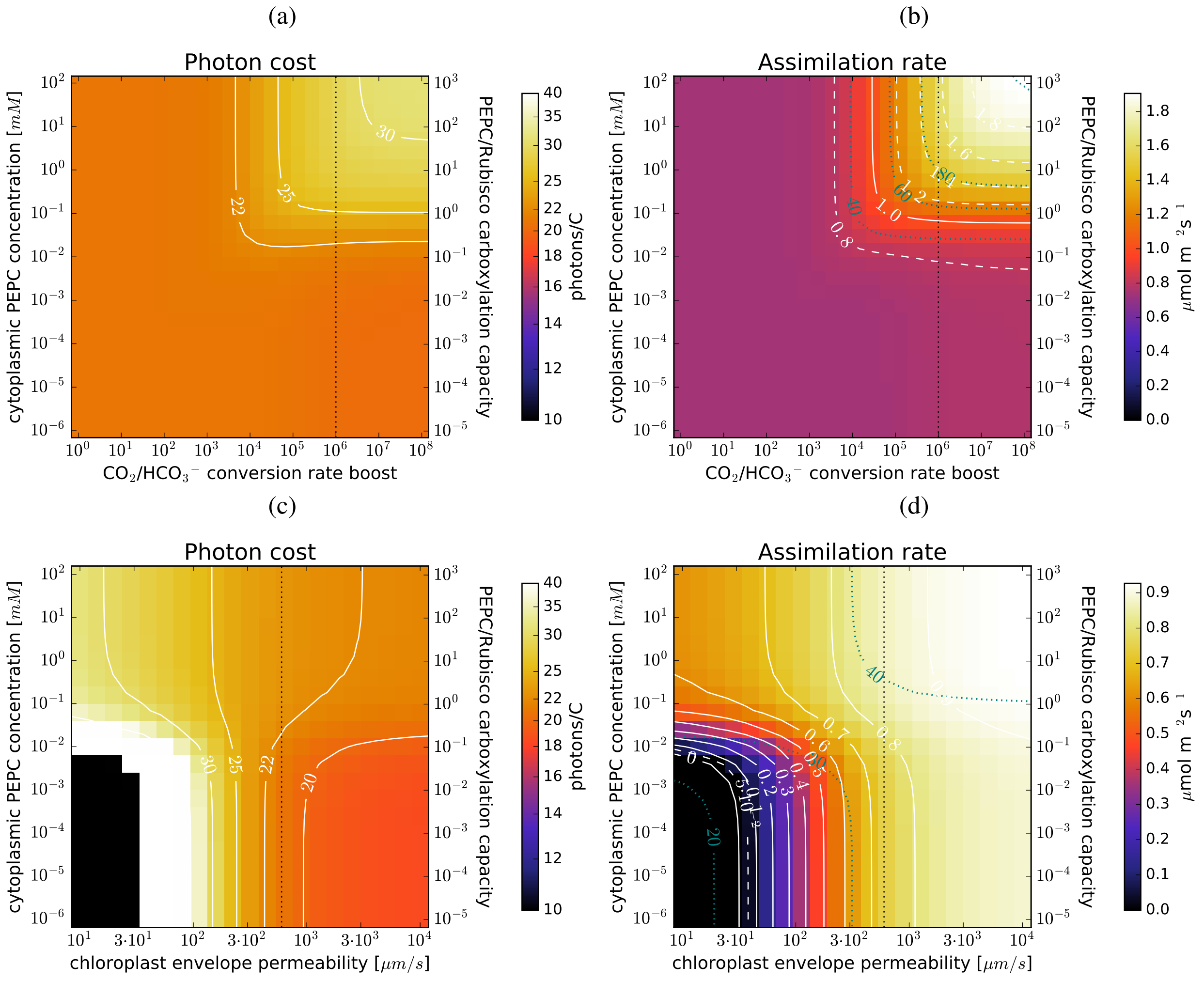

Figure G: Vacuole impact on C_4 photosynthesis: (a) and (b): The photon cost and the assimilation rate as functions of the vacuole membrane permeability σ_V and the cytoplasmic PEPC level (c_P), at σ_C = 200 μm/s, σ_P = 600 μm/s, and p_{CO_2} = 250 μbar. (c) and (d): The assimilation rate as a function of the drop of the vacuole (d_V) (which reduces the cytoplasmic volume) and the PEPC concentration (c) or the PEPC-to-RubisCO carboxylation capacity ratio (d). Parameters same as above, with $\sigma_V = 2\sigma_P = 1200\,\mu$m/s. The green lines in (b-d) mark the light-utilisation thresholds (in mol m^{-3}s^{-1}).

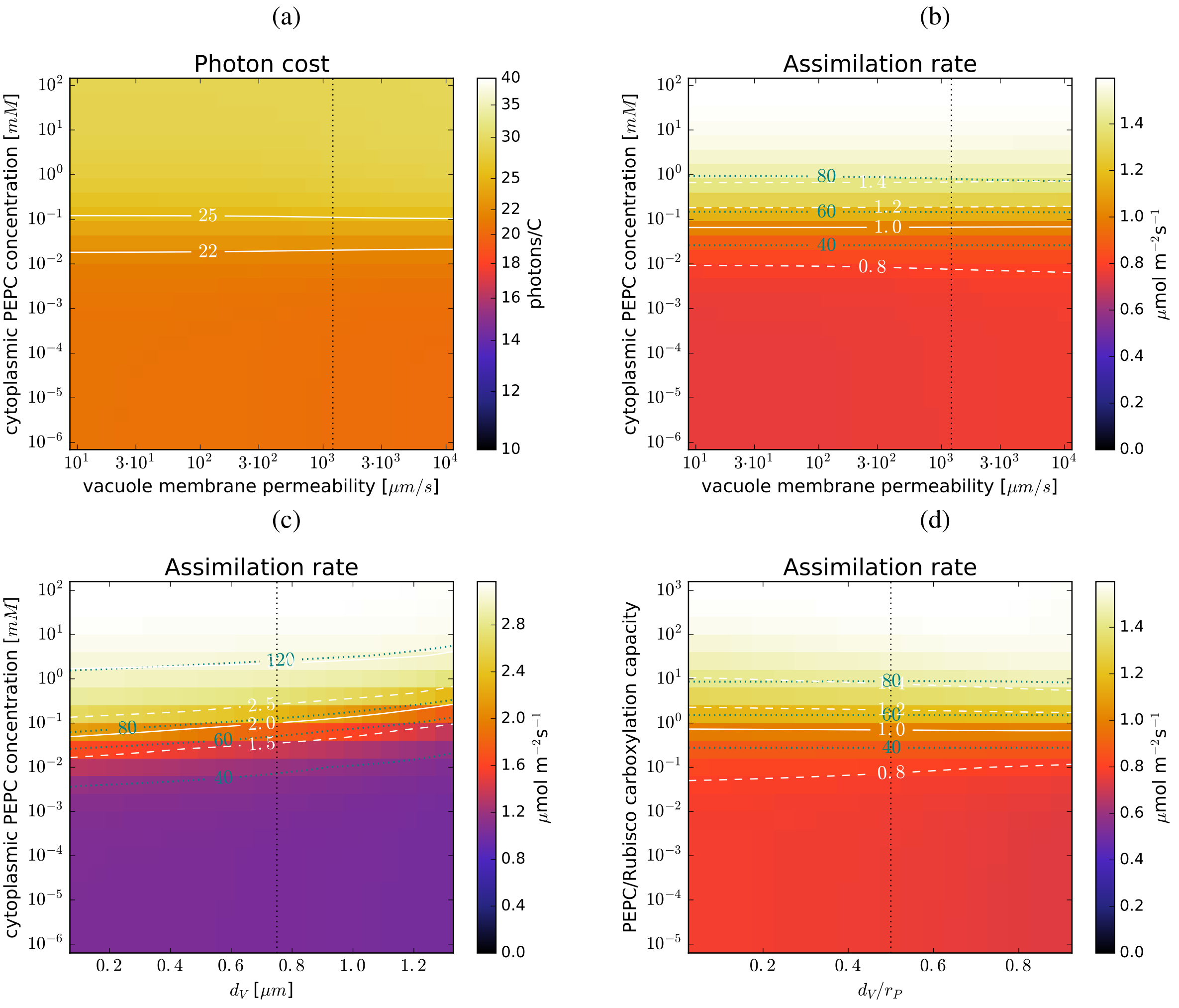

Figure H: Cell wall permeability and C_4 photosynthesis: (a) and (b): the photon cost and the net assimilation rate as functions of the combined permeability of the cell wall and plasmalemma (σ_C) and PEPC concentration in the cytoplasm (c_P), for the default parameter choice (Table 1 in the main text). The vertical dotted line marks the permeability used as default in other figures. The green lines in (b) mark the light-utilisation thresholds (in mol m^{-3}s^{-1}).

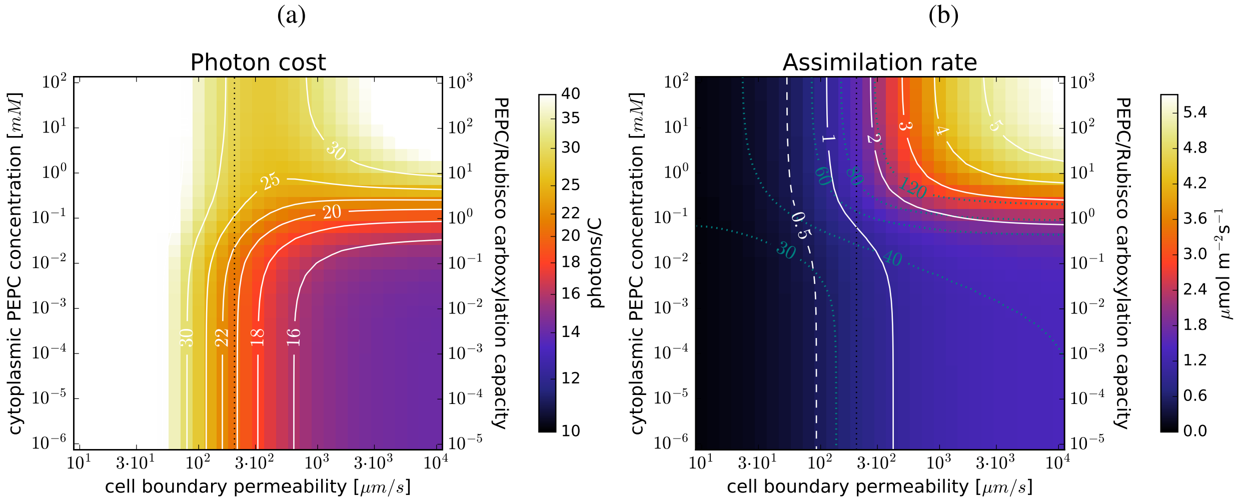

Figure I: Chloroplast envelope permeability to bicarbonate and C_4 photosynthesis: (a) and (b): the photon cost and the net assimilation rate as functions of the chloroplast envelope permeability to HCO_3^- and PEPC concentration in the cytoplasm, for the default parameter choice (Table 1 in the main text). The vertical dotted line marks the permeability used as default in other figures. The green lines in (b) mark the light-utilisation thresholds (in mol m^{-3}s^{-1}).

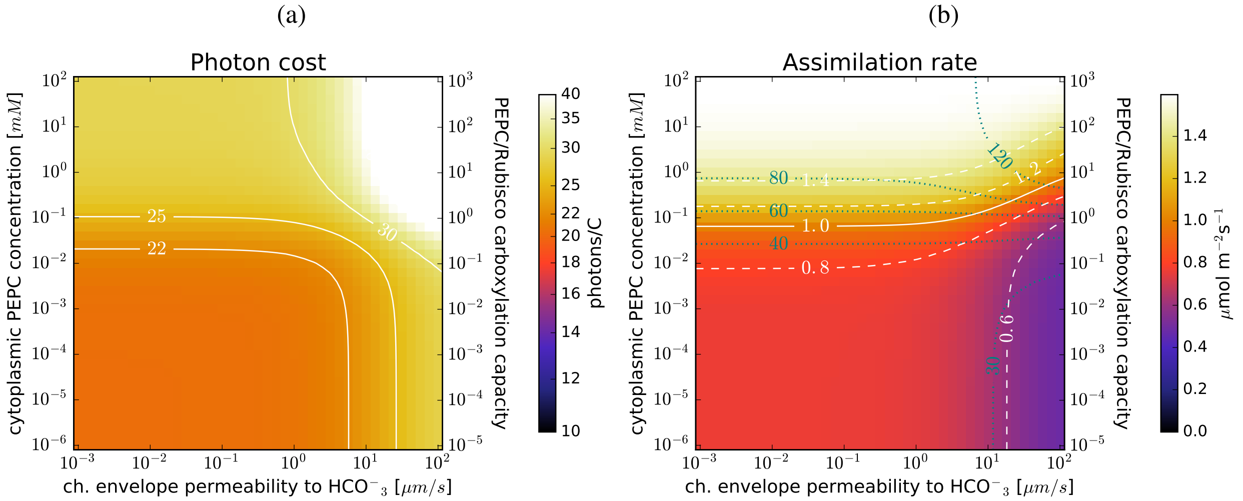

Figure J: Mesh precision: (a) and (b): the examples of meshing used for FEM integration at two different levels of precision. The mesh in (a) consists of 1084 triangle elements, while the mesh in (b) has 30492. The relative difference in integrated quantities (e.g. photon cost, carbon assimilation) in converged test runs on these two meshes is less than $2 \cdot 10^{-4}$. The meshing used to calculate the results presented in the paper used a medium level of precision, between the extremes of (a) and (b), with 6057 triangle elements in the default parameter case. (Note: the meshes in (a) and (b) do not represent the default geometry, but the the following: $r_P = 1\,\mu$m, $\phi_{plas/cell} = 25\%$, $d_{sep} = 0.03\,\mu$m, $\theta_{mem} = 0.01\,\mu$m, $d_V = 0.3\,\mu$m, and $h_V = 1.5\,\mu$m.)

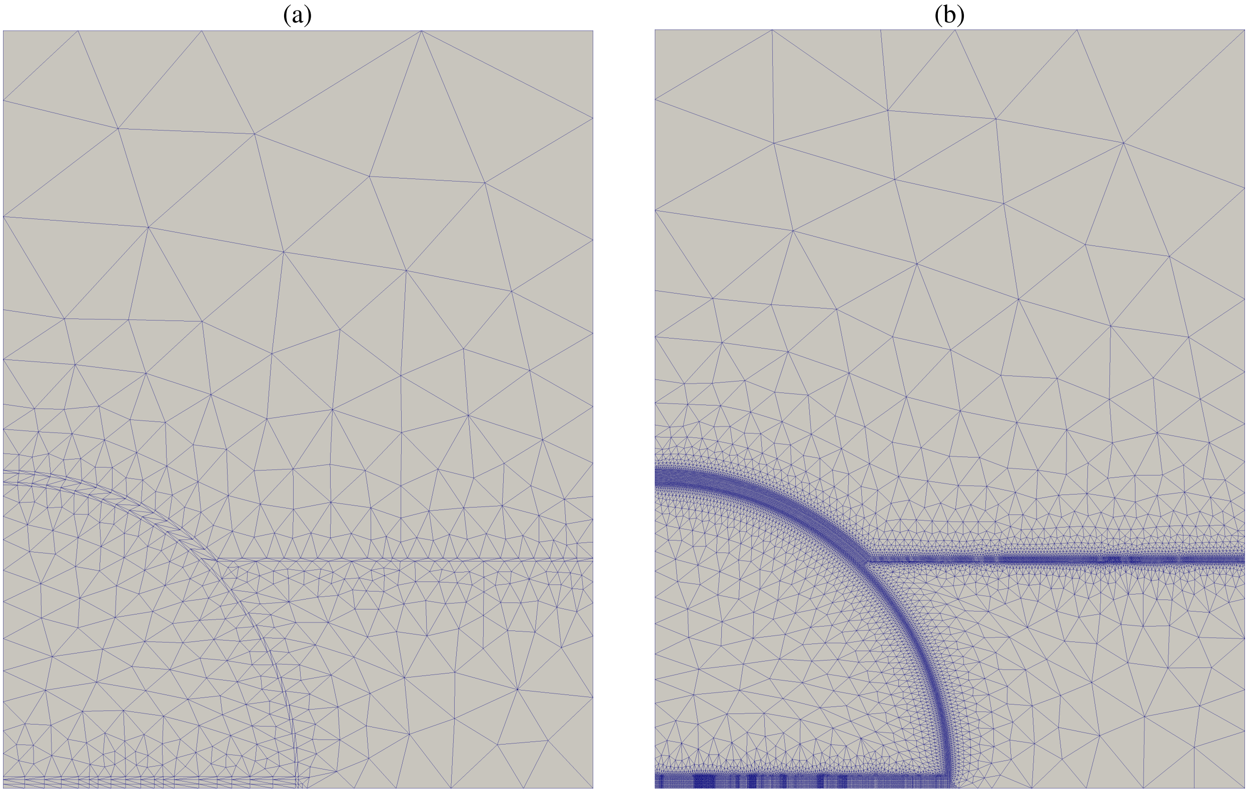

Figure K: Exemplary solutions: the top row - the concentration of $CO_2/O_2/HCO_3^-$; the middle and the bottom row - the radial and axial component of the respective diffusion current vectors. The left side shows a C_3 photosynthesis case, with the permeabilities $\sigma_P = \sigma_C = \frac{1}{2}\sigma_V = 10^3\,\mu$m/s, $p_{CO_2} = 200\,\mu$bar, and other parameters at their default values (Table 1 in the main text). The right side shows a C_4 photosynthesis case for the same choice of parameters and PEPC concentration $c_P = 1.1$ mM. The colour scale selected for concentrations differs between CO_2 (linear scale from 0 to $16\,\mu$M), O_2 (linear scale from 284 to $299\,\mu$M), and HCO_3^- (logarithmic scale from $8 \cdot 10^{-4}$ to $8 \cdot 10^{-1}$M), but is the same in the C_3 and the C_4 case. The current scales are same for all cases, with the orange colours representing a diffusion current component oriented toward the axis/bottom, and the blue colours representing a component oriented away from the axis/bottom.

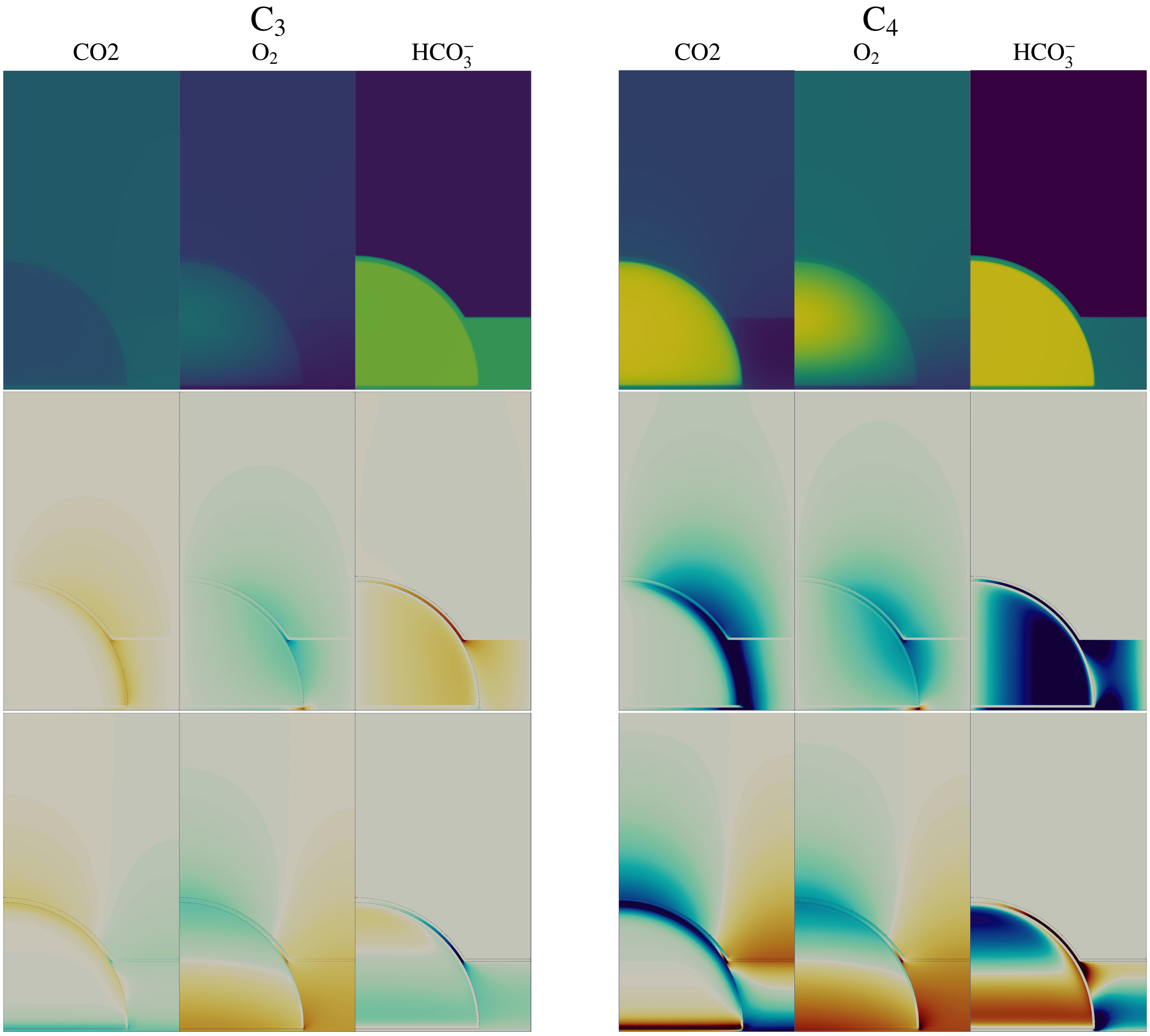

Supplement: S1 Figures — A document containing all the supplementary figures referenced in the text. (PDF) [file pcbi.1007373.s001.pdf]
