# Supplementary material for: Computational modelling predicts substantial carbon assimilation gains for C3 plants with a single-celled C4 biochemical pump
Source: PLoS Comput Biol. 2019 Sep 30;15(9):e1007373. doi: 10.1371/journal.pcbi.1007373 (PMC6786660; doi:10.1371/journal.pcbi.1007373)
Supplement: S1 Appendix — A more in-depth description of the model, with additional mathematical and technical details. (PDF) [file pcbi.1007373.s002.pdf]

Computational modelling predicts substantial carbon assimilation gains for C_3 plants with a single-celled C_4 biochemical pump

Ivan Jurić[a,*], Julian M. Hibberd[b], Mike Blatt[c], Nigel J. Burroughs[a,*]

[a] *Warwick Systems Biology Centre, University of Warwick, Coventry, CV4 7AL, UK*
[b] *Department of Plant Sciences, University of Cambridge, Cambridge, CB2 3EA, UK*
[c] *Laboratory of Plant Physiology and Biophysics, University of Glasgow, Glasgow, G12 8QQ, UK*

Supplementary Information

Mathematical outline of the model

We will use uppercase subscripts - O, C, and B - to denote the chemical species: O_2, CO_2, and HCO_3^- respectively. The lower case subscripts - p, c, v - will denote the three spatial regions considered: the *p*lastid, the *c*ytosol, and the *v*acuole. The same subscripts will also be used for the outer boundaries of these regions: the plastid envelope, the cell wall and membrane, and the vacuole membrane.

To find the gas currents under steady-state photosynthesis we need to solve the system of stationary diffusion-reaction equations for position-dependent concentrations of oxygen, carbon-dioxide, and bicarbonate - n_O, n_C, and n_B - satisfying appropriate boundary constraints and flux-balance conditions. The equations are of the form

$$D_i \nabla^2 n_i - r_i(\boldsymbol{n}) + s_i = 0 \tag{1}$$

where the index i stand for C, O, and B respectively. D_i is the compartment-dependent diffusion coefficient. r_i and s_i are the reaction and source terms. Both will depend on the location (the compartments, i.e. stroma, cytoplasm, and vacuole, or the intramembrane and intraenvelope spaces). The reaction term may in principle depend on the local value of any of the three concentrations, which we subsume into a 'vector' form $\boldsymbol{n} \equiv (n_O, n_C, n_B)$. The source terms are determined by flux-balance conditions to be addressed later. As all of the terms depend on the compartmental location, we introduce characteristic functions $\chi_p(\boldsymbol{r})$, $\chi_c(\boldsymbol{r})$, and $\chi_v(\boldsymbol{r})$, which are equal to one if the position vector, $\boldsymbol{r}$, is respectively within the plastid, cytoplasm, or vacuole, and zero otherwise. This way, we can specify the reaction terms as

$$r_O(\boldsymbol{n}(\boldsymbol{r}), \boldsymbol{r}) = \chi_p(\boldsymbol{r}) v_O c_R \frac{n_O(\boldsymbol{r})}{n_O(\boldsymbol{r}) + n_C(\boldsymbol{r}) K_O/K_C + K_O} \tag{2}$$

$$r_C(\boldsymbol{n}(\boldsymbol{r}), \boldsymbol{r}) = \chi_p(\boldsymbol{r}) v_C c_R \frac{n_C(\boldsymbol{r})}{n_C(\boldsymbol{r}) + n_O(\boldsymbol{r}) K_C/K_O + K_C} + v_{C\to B}(\boldsymbol{r}) n_C(\boldsymbol{r}) - v_{B\to C}(\boldsymbol{r}) n_B(\boldsymbol{r}) \tag{3}$$

*Corresponding authors. Emails: i.juric@warwick.ac.uk, n.j.burroughs@warwick.ac.uk.

$$r_B(\boldsymbol{n}(\boldsymbol{r}),\boldsymbol{r}) = \chi_c(\boldsymbol{r}) v_B c_P \frac{n_B(\boldsymbol{r})}{n_B(\boldsymbol{r}) + K_B} - v_{C\to B}(\boldsymbol{r}) n_C(\boldsymbol{r}) + v_{B\to C}(\boldsymbol{r}) n_B(\boldsymbol{r}) \quad (4)$$

where we used stationary Michaelis-Menten forms for the competitive reaction of RuBP-primed RubisCO (concentration c_R) with O_2 and CO_2 (see e.g. (1)), and for the reaction of the bicarbonate with PEPC (concentration c_P). $v_{C\to B}$ and $v_{B\to C}$ are the forward and backward reaction rates for the CO_2 to HCO_3^- interconversion. They will depend on the local pH value and on the presence or absence of the anhydrase. They are given by (2) as

$$v_{C\to B}(\boldsymbol{r}) = \eta_{CA}(\boldsymbol{r}) v_{C\to B\,(\text{base})}(\boldsymbol{r}) = \eta_{CA}(\boldsymbol{r}) \left(k_{CO_2} + k_{OH-K_w}/a_H(\boldsymbol{r})\right) \quad (5)$$

$$v_{B\to C}(\boldsymbol{r}) = \eta_{CA}(\boldsymbol{r}) v_{B\to C\,(\text{base})}(\boldsymbol{r}) = \eta_{CA}(\boldsymbol{r}) \left(k_d a_H(\boldsymbol{r}) + k_{HCO_3^-}\right) \quad (6)$$

$a_H(r)$ is the proton activity, given by the local pH, $a_H = 10^{-pH}\,\mathrm{M}$, and η_{CA} is the CA-dependent reaction boost factor. It is equal one where CA is absent (e.g. vacuole), and to a large number (10^6 by default) where CA is present (i.e. in the stroma and cytoplasm). The k-rates are taken from (2) as $k_{CO_2} = 0.037\,\mathrm{s}^{-1}$, $k_{OH-K_w} = 7.1 \cdot 10^{-11}\,\mathrm{Ms}^{-1}$, $k_d = 7.6 \cdot 10^4\,\mathrm{M}^{-1}\mathrm{s}^{-1}$, and $k_{HCO_3^-} = 1.8 \cdot 10^{-4}\,\mathrm{s}^{-1}$. The four rates correspond to two possible conversion pathways: CO_2+H_2O↔HCO_3^-+H^+ and CO_2+OH^-↔HCO_3^-. We do not allow for the CO_2-bicarbonate interconversion in the intramembrane and intraenvelope space (since it is an hydrophobic environment), so $v_{C\to B}$ and $v_{B\to C}$ are set to zero there.

Modelling the effect of carbonic anhydrase with a simple scaling factor is possible because the CA-mediated reaction is reversible and thus preserves the detailed balance of back and forth conversion rates (as long as H^+ and OH^- are in equilibrium). The scaling factor η_{CA} can be connected to CA concentration and its kinetic parameters as follows. The CA-mediated reaction can be expressed in a Michaelis-Menten form (e.g. (3))

$$v_{C\to B} n_C - v_{B\to C} n_B = \frac{c_{CA}\left(v'_{C\to B} K_B n_C - v'_{B\to C} K_C n_B\right)}{K_B K_C + K_B n_C + K_C n_B} \quad (7)$$

where v' are enzyme-substrate reaction rates, and K_B and K_C are characteristic HCO_3^- and CO_2 concentrations. In principle η_{CA} would thus depend on local n_C and n_B concentrations, as these terms appear in the divisor. However, with K_B and K_C in 10^0 M and 10^1 M range respectively (3), the divisor can be approximated as $K_B K_C$ and η_{CA} is approximately (for $\eta_{CA} \gg 1$)

$$\eta_{CA} = \frac{c_{CA} v'_{C\to B}}{K_C v_{C\to B\,(\text{base})}} = \frac{c_{CA} v'_{B\to C}}{K_B v_{B\to C\,(\text{base})}} \quad (8)$$

The source terms s_i reflect the release of O_2 and CO_2 as products of the relevant chemical pathways connected to photosynthesis. They are determined by the input of O_2, CO_2, and HCO_3^- as reactants in those pathways. We first define the cumulative fluxes J_{Calvin}, J_{phresp}, and J_{C4}.

$$J_{Calvin} = \int v_C c_R \frac{n_C(\boldsymbol{r})}{n_C(\boldsymbol{r}) + n_O(\boldsymbol{r}) K_C/K_O + K_C} \chi_p(\boldsymbol{r}) \mathrm{d}^3\boldsymbol{r} \quad (9)$$

$$J_{phresp} = \int v_O c_R \frac{n_O(\boldsymbol{r})}{n_O(\boldsymbol{r}) + n_C(\boldsymbol{r}) K_O/K_C + K_O} \chi_p(\boldsymbol{r}) \mathrm{d}^3\boldsymbol{r} \quad (10)$$

$$J_{C4} = \int v_{BCP} \frac{n_B(\boldsymbol{r})}{n_B(\boldsymbol{r}) + K_B} \chi_c(\boldsymbol{r}) \mathrm{d}^3\boldsymbol{r} \tag{11}$$

The source terms are

$$s_O(\boldsymbol{r}) = \chi_p(\boldsymbol{r}) \frac{J_{Calvin} + J_{phresp}}{V_p} \tag{12}$$

$$s_C(\boldsymbol{r}) = \chi_p(\boldsymbol{r}) \frac{J_{C4}}{V_p} + \chi_c'(\boldsymbol{r}) \frac{\frac{1}{2} J_{phresp}}{V_c'} \tag{13}$$

$$s_B = 0 \tag{14}$$

where V_i stand for the volumes of particular compartments, $V_i = \int \chi_i \mathrm{d}^3\boldsymbol{r}$. V_C' (and corresponding χ_C') stands for the part of the peripheral cytoplasmic space accessible to the mitochondria (excluding the narrow cytoplasmic gaps between the chloroplast and the cell and vacuole membranes).

The oxygen source term corresponds to the Hill reaction at the thylakoid, which is tied to RuBP carboxylation and oxygenation rates through NADPH flux balance: the production of NADPH via the linear electron transfer chain must match its consumption by the Calvin-Benson cycle and photorespiration. The CO_2 source term corresponds to the photorespiratory CO_2 release in the mitochondria and the release of CO_2 from the C_4 acid decarboxylation in the stroma.

The diffusion constant of a species in a particular compartment, D_i, is equal to the diffusion constant of that species in water $D_{i,aq}$ divided by the relative viscosity of the liquid filling the compartment with respect to water (as in (3)).

$$D_i(\boldsymbol{r}) = D_{i,aq} \left(\frac{\chi_p(\boldsymbol{r})}{\eta_p} + \frac{\chi_c(\boldsymbol{r})}{\eta_c} + \frac{\chi_v(\boldsymbol{r})}{\eta_v} \right) \tag{15}$$

However, within the tonoplast membrane and the chloroplast envelope, the diffusion coefficient is set to reflect the permeability of the particular barrier. For a barrier with thickness L and permeability σ, we have

$$D_{within} = L\sigma \tag{16}$$

Diffusion through the cell wall and plasmalemma is modelled by a boundary condition connecting the current density of CO_2 and O_2 perpendicular to the boundary surface with the difference between the local and equilibrium gas concentrations:

$$D_i \boldsymbol{\nabla} n_i(\boldsymbol{r} \in \partial\Omega) = \sigma_c \left(n_{i,eq} - n_i(\boldsymbol{r} \in \partial\Omega) \right) \hat{\boldsymbol{e}}_{\partial\Omega} = \sigma_c \left(H_i p_i - n_i(\boldsymbol{r} \in \partial\Omega) \right) \hat{\boldsymbol{e}}_{\partial\Omega} \tag{17}$$

Here i stands for O_2 or CO_2 (we assume HCO_3^- cannot cross the plasmalemma), $\partial\Omega$ is the cell boundary surface and $\hat{\boldsymbol{e}}_{\partial\Omega}$ is the unit vector perpendicular to that surface. σ_c is the combined permeability of the cell wall and the plasmalemma, while $n_{i,eq}$ is the stationary dissolved concentration of CO_2/O_2 in the thin wetting layer outside the cell wall, which is presumed to be in thermal equilibrium with the pressure, p_i, of the respective gas in the internal airspace: $n_{i,eq} = H_i p_i$ (H_i is the Henry constant). At other boundary surfaces we assume von Neumann boundary conditions, i.e. there is no current in or out of the simulated region

$$\boldsymbol{\nabla} n_i(\boldsymbol{r} \in \partial\Omega') = 0 \tag{18}$$

Light usage J_{photon} is evaluated from the overall fluxes in the energy consuming biochemical pathways.

$$J_{photon} = \varphi_{Calvin} J_{Calvin} + \varphi_{phresp} J_{phresp} + \varphi_{C4} J_{C4} \tag{19}$$

where φ_i stand for the photon cost of RuBP and PEP regeneration after each carboxylation or oxygenation event. The net photon cost of carbon assimilation is given by

$$\text{photon cost} = \frac{J_{photon}}{A} \tag{20}$$

where A is the net carbon assimilation rate (per plastid), which is determined by the competition of the Calvin and photorespiratory pathways:

$$A = J_{Calvin} - \frac{1}{2} J_{phresp} \tag{21}$$

The assimilation rate per cell surface is obtained by dividing A with the base of the simulated cylinder

$$A_{surf} = \frac{A}{\pi r_p^2 / \phi_{plas/cell}} \tag{22}$$

where $\phi_{plas/cell}$ is the chloroplast surface coverage (50% by default).

The required light use J_{photon} is also used to quantify the plastid-light harvesting capacity (LHC) which is expressed per stromal volume.

$$\text{LHC} = \frac{J_{photon}}{V_p} \tag{23}$$

It is compared to the maximal photosynthetically-active solar photon flux, j_{PAPF}, by considering the fraction of this flux that would be absorbed by an array of plastids in the peripheral cytoplasmic layer of a cell (see Fig. 1d in the main text), at the default surface coverage ratio, $\phi_{plas/cell}$

$$\text{absorbed fraction} = \frac{J_{photon}}{\pi r_p^2 / \phi_{plas/cell}} : j_{PAPF} \tag{24}$$

We have posed the model in a very general way as a system of nonlinear partial integro-differential equations in three dimensions. In reality we seize the advantage of the postulated cylindrical symmetry of the system. The resulting problem, which is effectively two-dimensional[1], is solved iteratively by a finite element method on a prespecified simplex mesh. The mesh is algorithmically constructed to follow the natural boundaries of the simulated system (i.e. the internal and external surface of the envelope and the tonoplast membrane). We use DUNE/PDELab libraries with BCGS solver on P_2 elements (4; 5). As nonlinear PDE's require iterative solving, there is a natural way to include our integrative flux-balance conditions by updating the source terms with each iteration. The ability of the mesh to accurately capture the PDE solution was tested by comparing the typical results for meshes with different levels of

[1]Some care must be taken in converting the three-dimensional Laplacian from cylindrical coordinates into a two-dimensional Laplacian. The three-dimensional Laplacian is $\nabla^2 = \frac{1}{r}\frac{\partial}{\partial r}\left(r\frac{\partial}{\partial r}\right) + \frac{1}{r^2}\frac{\partial^2}{\partial \varphi^2} + \frac{\partial^2}{\partial z^2}$, which (for a cylindrically symmetric case with $\frac{\partial^2}{\partial \varphi^2} = 0$) simplifies into $\nabla^2 = \frac{1}{r}\frac{\partial}{\partial r} + \nabla^2_{2D}$ where $\nabla^2_{2D} = \frac{\partial^2}{\partial r^2} + \frac{\partial^2}{\partial z^2}$ is the two-dimensional Laplacian used on the finite-element-method mesh. The $\frac{1}{r}\frac{\partial}{\partial r}$ term is an additional 'pseudo-source' term that appears in the 2D formulation.

precision (Fig J in S1 Figures). Upon achieving convergence, the integrated currents (J_{Calvin}, J_{phresp}, and J_{C4}) at different mesh precisions differed by less than 2 parts in 10^4. Fig K in S1 Figures shows exemplary solutions with and without an active C_4 cycle.

The light-limited operation can be simulated by also evaluating the energy usage J_{photon} during each iteration and scaling the concentration of RubisCO and PEPC, if the usage exceeds some capping threshold J_{cap}.

$$c_{R/P}^{n+1} = \min\left(c_{R/P(\text{base})}, c_{R/P}^{n} \frac{J_{cap}}{J_{photon}^{n}} \right) \tag{25}$$

where we denoted the iteration number in the superscript.

Model implementation

For iterative solving of the system of coupled integral and partial-differential equations, a custom C++ code was assembled, utilising the publicly avaliable Dune/PDElab framework for finite-element-method integration. Pre-integration mesh construction and subsequent data analysis and visualisation were done by custom Python scripts relying on the publicly avaliable NumPy/SciPy and Matplotlib libraries.

References

[1] Witzel F, Götze J, Ebenhöh O. Slow deactivation of ribulose 1,5-bisphosphate carboxylase/oxygenase elucidated by mathematical models. The FEBS Journal. 2010;277(4):931–950. doi:10.1111/j.1742-4658.2009.07541.x.

[2] Johnson KS. Carbon dioxide hydration and dehydration kinetics in seawater1. Limnol Oceanogr. 1982;27(5):849–855. doi:10.4319/lo.1982.27.5.0849.

[3] Tholen D, Zhu XG. The Mechanistic Basis of Internal Conductance: A Theoretical Analysis of Mesophyll Cell Photosynthesis and CO2 Diffusion. Plant Physiol. 2011;156(1):90–105. doi:10.1104/pp.111.172346.

[4] Blatt M, Burchardt A, Dedner A, Engwer C, Fahlke J, Flemisch B, et al. The Distributed and Unified Numerics Environment, Version 2.4. University Library Heidelberg; 2016.

[5] Alkämper M, Dedner A, Klöfkorn R, Nolte M. The DUNE-ALUGrid Module. Archive of Numerical Software. 2016;4(1):1–28. doi:10.11588/ans.2016.1.23252.
